# Understanding the Performance of Deep Computer Vision Models: A Symbolic Regression Approach to Accuracy and Latency Prediction

**DOI:** 10.3390/s26134093

**Published:** 2026-06-27

**Authors:** Divyesh Rameshbhai Dhanani, Faraz Kayani, Saif U Din, Alice Arslanian, Dmitry Ignatov, Radu Timofte

**Affiliations:** Computer Vision Lab, Center for Artificial Intelligence and Data Science (CAIDAS) and Institute of Computer Science (IFI), University of Würzburg, John-Skilton-Straße 4a, 97074 Würzburg, Germany; faraz.kayani@stud-mail.uni-wuerzburg.de (F.K.); saif.saif-u-din@stud-mail.uni-wuerzburg.de (S.U.D.); alice.arslanian@stud-mail.uni-wuerzburg.de (A.A.); radu.timofte@uni-wuerzburg.de (R.T.)

**Keywords:** edge AI, edge hardware, deep learning, computer vision, inference latency, accuracy prediction, symbolic regression, distance correlation, neural architecture, interpretable machine learning

## Abstract

Deploying deep vision models on edge hardware requires understanding how architecture and training hyperparameters jointly determine accuracy and inference latency, yet these relationships remain poorly characterized in a systematic, data-driven manner. This paper presents a two-stage statistical framework providing interpretable, closed-form insights into both. In the first stage, we apply distance correlation (dCor) and the maximal information coefficient (MIC) across seven image-classification datasets, revealing that batch size and total layer count are the strongest universal accuracy predictors (mean dCor: 0.228 and 0.174), while learning rate achieves the highest MIC (0.226), reflecting a non-monotonic relationship with accuracy. In the second stage, PySR symbolic regression (representing, to our knowledge, the first application to cross-dataset vision model accuracy prediction) derives compact, interpretable formulas. Dataset-specific models achieve $R^{2}$ from 0.20 to 0.45; a universal model achieves a mean leave-one-dataset-out $R^{2}$ of 0.23, remaining strictly positive on all held-out datasets, whereas ordinary linear regression collapses to $R^{2}=-0.71$. We further derive device-specific inference latency formulas for CPU, GPU, and NPU, outperforming classical baselines by 6.7×–14.8× in $R^{2}$ and confirming fundamental device heterogeneity. Together, these results offer interpretable surrogate models for screening deep vision architectures under accuracy and latency constraints in edge deployment.

## 1. Introduction

Deploying deep neural networks on resource-constrained edge hardware platforms is one of the main challenges of applied machine learning [1,2]. These platforms include embedded devices such as mobile SoCs, microcontrollers, and dedicated AI accelerators deployed on drones, autonomous vehicles, and IoT nodes. Unlike cloud deployment, inference on edge hardware must satisfy hard limits on memory footprint, floating-point operations per second (FLOPs), and energy budget, all while maintaining acceptable accuracy on the target task. In modern edge deployments, deep computer vision networks process visual data captured by onboard sensors, and the accuracy and latency characteristics of these models directly determine the reliability and throughput achievable by the encompassing system.

Practitioners typically address this tension through one of two approaches: (i) they apply compression techniques such as pruning, quantization, or knowledge distillation [3] to an existing high-accuracy model, or (ii) they design architectures from the ground up with efficiency as a first-class objective [4,5]. Both approaches require an understanding of howarchitectural decisions and training hyperparameters influence final accuracy. Without such understanding, model selection and configuration remain largely empirical: trial-and-error processes guided by intuition rather than principled relationships.

Prior work has made progress on isolated aspects of this problem. Scaling laws relate model size and dataset size to loss [6,7]. Neural architecture search (NAS) provides a systematic but computationally expensive search strategy [8]. Theoretical studies have derived bounds on how depth and width affect approximation capacity [9,10]. However, these contributions share a common limitation: they are typically derived for a single architecture family, a single benchmark, or a narrow range of training configurations. They rarely produce formulas that are simultaneously (a) empirically derived from diverse experimental data, (b) expressed in closed symbolic form, and (c) validated across multiple datasets.

This paper addresses that gap. We propose a pipeline that combines nonparametric statistical correlation analysis with evolutionary symbolic regression [11], which has previously proven effective at recovering closed-form physical laws directly from experimental data, to produce interpretable accuracy-prediction formulas for deep vision models. Specifically, our contributions are:

1. **Feature–accuracy correlation study.** We systematically measure dCor and MIC between eleven architectural and hyperparameter features and model accuracy across seven image-classification datasets and three training checkpoints (epochs 1, 5, and 50), with full multiple-testing correction (Benjamini–Hochberg FDR at α=0.05).
2. **Dataset-specific symbolic formulas.** For each dataset and epoch, we use PySR to derive a Pareto-optimal set of accuracy-prediction equations, validated by five-fold cross-validation, with $R^{2}$ values up to 0.45. The derived formulas assume depth-based capacity scaling and may not generalize to architectures whose primary scaling dimension is width or channel count, such as EfficientNet [12] and MobileNet [5]; this limitation is analyzed in Section 9.
3. **Universal formula.** We pool data from all seven datasets, apply a z-score normalization to remove dataset-level offsets, and derive a single universal formula that generalizes to unseen datasets via LODO cross-validation, with a mean LODO $R^{2}$ of 0.23. Although pooled in-sample $R^{2}$ is modest and gradient boosting attains higher scores on the same features, the formula’s principal value is interpretability and robust cross-dataset ranking: on LODO, Linear regression collapses to $R^{2}=-0.71$ when CIFAR-10 is held out, while the symbolic formula stays positive on all seven held-out datasets (Section 7.4).
4. **Comparative analysis.** We position our approach against the existing scaling-law and architecture-analysis literature, identifying the advantages of symbolic regression in interpretability and cross-domain generalizability.
5. **Inference latency estimation (complementary application).** We apply the same symbolic-regression methodology (but not the same features, targets, or evaluation protocol) to hardware inference latency as a complementary case study. Using a dataset of 1161 measured inference samples per device (CPU, GPU, and NPU) collected under both FP32 and INT8 precision configurations on CIFAR-10, we apply PySR to discover device-specific closed-form latency formulas from architectural features. Validated against four classical baselines (FLOPs-only linear regression, multi-feature linear regression, the PALEO efficiency model [13], and the analytical Roofline model [14]), our symbolic formulas achieve $R^{2}$ improvements of 6.7×–14.8× and empirically demonstrate that inference latency is fundamentally device-heterogeneous: a single pooled formula yields negative $R^{2}$ on held-out millisecond predictions, confirming the necessity of device-specific modeling.

### Scientific Contribution vs. Practical Results

The methods used in this paper, dCor, MIC, and PySR, are established tools from statistics and symbolic regression. The scientific contribution lies not in introducing a new algorithm, but in three distinct aspects. First, the combined methodological pipeline applies dual-measure nonparametric screening to select features and then uses staged symbolic regression with rigorous five-fold cross-validation and leave-one-dataset-out evaluation, a protocol not previously applied to the problem of cross-dataset vision model accuracy prediction. Second, the empirical finding that this pipeline yields strictly positive cross-dataset generalization under LODO while ordinary linear regression collapses to $R^{2}=-0.71$ is a new, reproducible result about the structure of accuracy prediction across diverse benchmarks. Third, the discovery of interpretable closed-form formulas from a dataset of over 246,000 records spanning seven benchmarks constitutes a reusable empirical finding independent of the specific tools used. The practical outputs, the formulas themselves, follow from applying this framework to the data and carry direct deployment value; the methodological framework and the cross-dataset empirical findings are the primary scientific contribution.

The remainder of the paper is structured as follows: Section 2 reviews related work. Section 3 describes the datasets, features, and methodology, including the inference latency modeling pipeline. Section 4 presents the experimental setup. Section 5 reports per-dataset correlation and symbolic regression results. Section 6 presents the device-specific inference latency formulas and their baseline comparisons. Section 7 presents the universal accuracy model. Section 8 compares our approach with existing work. Section 9 discusses the findings and limitations. Section 10 concludes. Appendix A describes the convergence analysis used to select optimal PySR iteration counts and provides extended baseline comparison tables for inference latency.

## 2. Related Work

### 2.1. Scaling Laws and Model Size

The idea that neural network performance follows predictable mathematical laws with respect to model size and training resources has a long history, but was placed on a rigorous empirical footing by Kaplan et al. [6], who showed that language model test loss obeys consistent power-law behavior with respect to parameter count *N*, dataset size *D*, and compute budget *C*:

(1) $$L\left(N\right)\approx {\left(\frac{N_{c}}{N}\right)}^{\;\alpha_{N}},\;L\left(D\right)\approx {\left(\frac{D_{c}}{D}\right)}^{\;\alpha_{D}},\;L\left(C\right)\approx {\left(\frac{C_{c}}{C}\right)}^{\;\alpha_{C}}.$$

Bahri et al. [7] subsequently provided theoretical grounding for these empirical laws by identifying four distinct scaling regimes depending on the relative sizes of model and dataset, and showing that scaling exponents are universal in variance-limited regimes but architecture-dependent in resolution-limited regimes. While these scaling laws are influential, they were derived primarily for language models and focus on the asymptotic large-scale regime. Our work complements them by targeting the moderate-scale, multi-task setting characteristic of edge deployment.

### 2.2. Architectural Dependencies: Depth, Width, and Capacity

A foundational empirical insight is that network depth improves accuracy up to a point. Simonyan and Zisserman [15] demonstrated that increasing VGG depth from 11 to 19 layers consistently improves ImageNet classification accuracy. He et al. [16] extended this observation to very deep networks (100+ layers) by introducing residual connections:

(2) $$x_{l+1}=x_{l}+F\left(x_{l},\;W_{l}\right),$$

showing that skip connections alleviate the optimization difficulties that otherwise prevent deep networks from outperforming shallower ones. Zagoruyko and Komodakis [17] showed that widening residual networks can match or exceed the accuracy of deeper networks, and Tan and Le [12] showed that jointly scaling depth, width, and resolution under a compound coefficient outperforms scaling any single dimension. Golubeva et al. [18] confirmed that width remains beneficial even under fixed parameter budgets. Nguyen et al. [19] further observed that wide and deep models, despite reaching similar accuracy, fail on different input examples, a phenomenon with direct implications for ensemble and compression strategies. From the theoretical side, Safran and Shamir [20] proved that shallow networks may require exponentially more neurons than deep networks to achieve the same approximation quality.

These results collectively establish that both depth and width matter, but their interaction with training hyperparameters and task difficulty is complex. Chen and Tsou [21] investigated depth, width, and cardinality jointly for remote sensing tasks and found that tuning all three together outperforms tuning any single factor. Our dCor results confirm and quantify this complexity across a much more diverse task set.

### 2.3. Hyperparameter Dependencies

Smith [22] conducted one of the first systematic empirical studies of learning rate, batch size, momentum, and weight decay, showing that these hyperparameters interact strongly and that finding their joint optimum is important for performance. Li et al. [23] further showed that the optimal learning rate is a non-monotonic function of batch size for adaptive optimizers, with a peak at a noise-scale-dependent critical batch size $B_{\mathrm{noise}}$:

(3) $$\epsilon_{\mathrm{opt}}\left(B\right)\approx \frac{\sqrt{\frac{B_{\mathrm{noise}}}{2\pi }}}{\frac{1}{2}\;\left(\sqrt{\frac{B_{\mathrm{noise}}}{B}}+\sqrt{\frac{B}{B_{\mathrm{noise}}}}\right)}\cdot \frac{\sum_{i}\frac{\mu_{i}^{2}}{\sigma_{i}}}{\sum_{i}H_{i,i}}.$$

Our MIC analysis captures the non-monotonic nature of the learning rate–accuracy relationship (MIC =0.226 vs. Spearman |ρ|=0.312), confirming that linear correlation measures are insufficient for a complete picture.

### 2.4. Non-Monotonic Behavior and Double Descent

Classical bias-variance theory [24] predicts that test error decreases monotonically as model capacity increases until overfitting begins. Nakkiran et al. [25] challenged this with the empirical “double descent” phenomenon: test error first decreases, then increases near the interpolation threshold, and then decreases again for overparameterized models. Lafon and Vigogna [26] provide theoretical analysis explaining these transitions. These non-monotonic relationships motivate our use of dCor and MIC rather than Pearson or Spearman correlation, since both dCor and MIC capture arbitrary statistical dependencies including non-monotonic ones.

### 2.5. Regularization Effects

Regularization techniques modify the relationship between network capacity and accuracy in ways that matter for edge deployment. Batch normalization [27] improves training stability by normalizing intermediate activations:

(4) $${\hat{x}}_{i}=\frac{x_{i}-\mu_{B}}{\sqrt{\sigma_{B}^{2}+\varepsilon }},\;y_{i}=\gamma \;{\hat{x}}_{i}+\beta ,$$

which allows higher learning rates and speeds up convergence. Dropout [28] randomly disables neurons during training, which improves generalization by reducing co-adaptation. Our dropout count feature captures the cumulative use of dropout across network layers, and our results show it ranks among the top five features by mean dCor (0.164).

### 2.6. Computational Cost and Inference Latency Prediction

FLOPs are a widely used proxy for computational cost, but they systematically fail to predict actual hardware inference latency. Bouzidi et al. [29] show that latency on mobile and edge hardware is determined as much by memory access patterns, cache behavior, and instruction-level parallelism as by raw FLOPs. Zhang et al. [30] build regression models for latency prediction on diverse edge devices, showing that multi-feature models outperform FLOPs-only baselines and that device heterogeneity requires device-specific treatment, a finding our results corroborate and extend.

Analytical performance models offer an alternative approach. The Roofline model [14] frames latency as determined by the lesser of peak compute throughput and memory bandwidth:

(5) $$t=\frac{F}{\min(P_{\mathrm{peak}},\;I\cdot \mathrm{BW})},$$

where I=F/MAC is arithmetic intensity. Despite its theoretical grounding, the Roofline model assumes fixed hardware parameters that rarely hold for real deep learning workloads, where thermal throttling, shared memory bandwidth, and dynamic voltage scaling all introduce systematic deviations. The PALEO model [13] assumes constant effective FLOPS utilization per device and precision level, an assumption that fails across heterogeneous CNN architectures with different cache and pipeline behaviors.

Hardware-aware neural architecture search (NAS) methods, including ProxylessNAS [31], FBNet [32], and TVM-based approaches [33], learn latency predictors as part of the NAS loop, typically using black-box lookup tables or lightweight regression models on operator-level timing. While highly accurate within their training distribution, these predictors are architecture-specific, require expensive device profiling, and produce no interpretable closed-form equation. *μ*NAS [34] extends this to microcontroller targets but faces the same interpretability limitation.

Quantization precision is a further driver of inference latency that classical models poorly capture. Jacob et al. [35] demonstrate that INT8 inference activates qualitatively different hardware execution paths (dedicated integer multiply-accumulate units, different instruction-cache footprints, and distinct memory bandwidth utilization) compared with FP32. A single efficiency scalar therefore cannot represent both precision regimes simultaneously.

A key gap across this literature is the absence of interpretable, closed-form, device-specific latency formulas derived empirically from diverse neural architectures. Prior work either (i) uses black-box latency lookups that offer no symbolic insight, (ii) applies analytical models that assume hardware properties rarely achieved in practice, or (iii) fits linear regression models that cannot capture the non-linear, exponential scaling of latency with architecture parameters. We address this gap by applying PySR symbolic regression to discover Pareto-optimal closed-form latency equations for CPU, GPU, and NPU devices, trained on FP32 and INT8 measurements across 500+ neural network architectures on CIFAR-10. Our inclusion of both FLOPs and parameter count as features captures complementary aspects of model computational cost, while log-transformed targets ensure stable regression across the wide dynamic range of measured latencies.

### 2.7. Limitations of Existing Approaches

Despite this progress, Zhang et al. [36] demonstrated that deep neural networks can perfectly memorize randomly labeled data while still generalizing on real data, a result that complicates the relationship between model capacity and accuracy. Graph-theoretic approaches attempt to link network topology to generalization using structural metrics, but remain difficult to interpret and generalize across tasks.

A key gap across this literature is the absence of compact, symbolic, cross-dataset formulas. Prior work either (i) identifies which features matter (correlation studies) without expressing the relationship mathematically, (ii) derives scaling laws for large language models that do not transfer to edge vision tasks, or (iii) produces black-box performance predictors (NAS surrogates, neural predictors) that lack interpretability. Our approach is the first to combine nonparametric correlation screening with symbolic regression to produce Pareto-optimal, human-readable accuracy formulas validated across a diverse benchmark collection. A review of the documented PySR application literature [37], which spans physics, astrophysics, fluid dynamics, and biology, reveals no prior application of PySR or analogous symbolic regression frameworks to cross-dataset vision model accuracy prediction; this paper is, to our knowledge, the first to do so.

## 3. Methodology

### 3.1. Overview

**Figure 1:**
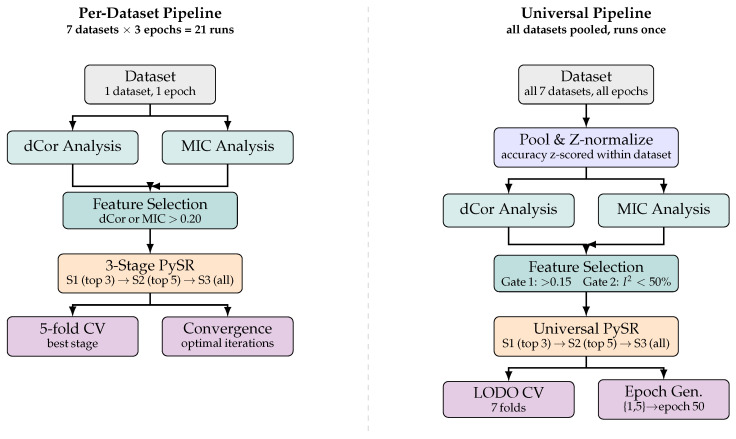
Combined pipeline overview. (**Left**) Per-Dataset Pipeline:for each of the 21 dataset–epoch combinations (7 datasets × 3 epochs), raw accuracy is fed into parallel dCor and MIC correlation screening, followed by feature selection (threshold > 0.20), three-stage PySR symbolic regression, and optional 5-fold cross-validation (CV) and convergence analysis. (**Right**) Universal Pipeline: all datasets are pooled and z-normalized (accuracy z-scored within each dataset), then passed through correlation screening (Gate 1: dCor/MIC > 0.15; Gate 2: $I^{2}<50\%$), universal three-stage PySR, and validated via leave-one-dataset-out (LODO) cross-validation and epoch generalization.

Our pipeline consists of four stages: (1) data collection and splitting, (2) correlation analysis, (3) dataset-specific symbolic regression, and (4) universal pooled regression. Figure 1 provides a schematic overview.

### 3.2. Datasets and Features

We assembled experimental records from seven image-classification benchmarks: **CelebA-Gender** [38] (binary facial attribute classification), **CIFAR-10** and **CIFAR-100** [39] (10-class and 100-class object recognition), **ImageNette** [40] (10-class subset of ImageNet), **MNIST** [41] (handwritten digit recognition), **Places365** [42] (scene classification), and **SVHN** [43] (street view house number recognition). For each dataset, multiple deep neural network architectures were trained with diverse hyperparameter configurations, and accuracy measurements were collected at training epochs 1, 5, and 50.

Each training run is described by eleven features, summarized in Table 1.

Dataset-level sample counts at epoch 50 are: CelebA-Gender: 24,310; CIFAR-10: 2039 (subset at epoch 50); CIFAR-100: 387; ImageNette: 32,381; MNIST: 38,216; Places365: 2875; SVHN: 41,366. The pooled dataset used for universal analysis (symbolic regression training and LODO cross-validation) contains approximately 246,000 records across all datasets and epochs. An independent subset of 49,770 records drawn from the LEMUR 2 corpus [44] (distinct from the ≈246,000-record symbolic regression training pool described in Section 3.2 and Section 7.1) is used separately for calibration and confidence-interval estimation in Section 7.3.

#### Relevance to Edge Hardware Deployment

The seven benchmarks are standard image-classification datasets and do not include raw sensor streams such as thermal imagery or LiDAR point clouds. They are used because they provide a large, diverse, and reproducible corpus of architecture–hyperparameter–accuracy triples, which is the input required by the statistical and symbolic regression pipeline. The findings of this study, specifically which architectural and hyperparameter features predict accuracy and how they interact, are properties of the trained models and therefore transfer to any classification task those models might be deployed on, including tasks on embedded vision hardware. The latency results (Section 3.6) are measured directly on CPU, GPU, and NPU hardware, and therefore represent real edge deployment conditions without this indirect connection. We acknowledge that extending the accuracy analysis to datasets collected from embedded or automotive sensors would strengthen the practical grounding of the findings, and we identify this as a direction for future work.

### 3.3. Correlation Analysis

We apply two complementary nonparametric dependence measures to each feature–accuracy pair.

#### 3.3.1. Distance Correlation (dCor)

Distance correlation [45] measures general statistical dependence, including nonlinear relationships. It is defined for random vectors *X* and *Y* as

(6) $$\mathrm{dCor}\left(X,Y\right)=\sqrt{\frac{{\mathrm{dCov}}^{2}\left(X,Y\right)}{\sqrt{{\mathrm{dCov}}^{2}\left(X,X\right)\cdot {\mathrm{dCov}}^{2}\left(Y,Y\right)}}},$$

where ${\mathrm{dCov}}^{2}\left(X,Y\right)$ is the distance covariance, computed from pairwise distance matrices. Importantly, dCor(X,Y)=0 if and only if *X* and *Y* are statistically independent, so it is a universal dependence measure. dCor values lie in [0,1], with larger values indicating stronger dependence.

We compute statistical significance using the analytical *t*-test approximation of Székely and Rizzo [46], which avoids computationally expensive permutation testing. All *p*-values are corrected jointly across all feature–dataset–epoch combinations using the Benjamini–Hochberg false discovery rate (BH-FDR) procedure at α=0.05 [47]. Distance correlation is computed using the AVL O(nlogn) algorithm [48], which produces results identical to the naive $O(n^{2})$ algorithm. Spearman rank correlation is retained as a linear reference baseline.

#### 3.3.2. Maximal Information Coefficient (MIC)

The maximal information coefficient [49] captures the maximum normalized mutual information achievable by any partitioning of the (X,Y) space into a grid, over all grids with at most $n^{\alpha_{\mathrm{MINE}}}$ cells:

(7) $$\mathrm{MIC}\left(X,Y\right)=\max_{ab\;\leq \;n^{\alpha }}\frac{I^{*}\left(X,Y;\;a,b\right)}{\log\min(a,b)},$$

where $I^{*}\left(X,Y;\;a,b\right)$ is the maximum empirical mutual information over a×b grids. We use the MINE library with α=0.6 and c=15 (standard settings from the original paper). Statistical significance is assessed by a permutation test with N=200 shuffles per feature pair, with BH-FDR correction.

While dCor is theoretically grounded with an efficient analytical test, MIC provides a finer-grained sensitivity to non-monotonic and multi-modal relationships. The two measures are complementary: a feature with high MIC but low Spearman |ρ| indicates a non-monotonic but real relationship with accuracy.

#### 3.3.3. Feature Selection for Symbolic Regression

A feature is considered statistically significant for symbolic regression input if it exceeds a threshold of 0.20 in either dCor or MIC. Significant features are ranked by dCor and fed into a staged PySR process (Section 3.4).

### 3.4. Symbolic Regression with PySR

PySR [50] is an open-source, evolutionary symbolic regression framework based on simulated annealing and genetic algorithms. It searches the space of mathematical expressions built from a user-specified set of operators and produces a Pareto front of solutions trading off formula complexity against prediction loss.

We configure PySR with:

- Binary operators: {+,−,×,÷,x^}
- Unary operators: $\left\{\sqrt{\cdot },\;\log,\;|\cdot |,\;\exp,\;{\left(\cdot \right)}^{2}\right\}$
- Maximum formula size: 30 nodes
- Number of populations: 30
- Random seed: 42 (fully deterministic)

We run a three-stage pipeline designed to balance expressivity with parsimony, as summarized in Table 2.

We select the best equation per stage by its $R^{2}$ on the full training set, and take the best stage overall as the one achieving the highest $R^{2}$. We then perform five-fold cross-validation (CV) on the best stage to estimate generalization: for each fold, we train one PySR model using the dataset-optimal iteration count (50–500, determined by convergence analysis; see Appendix A) and evaluate $R^{2}$ on the held-out fold. We also evaluate the reference equation (best model from the full training set) on each validation fold.

### 3.5. Universal (Pooled) Analysis

For the universal model, all seven datasets and three epochs are combined. To remove dataset-level accuracy offsets while preserving epoch-wise trends, accuracy is z-scored within each dataset (across all epochs for that dataset):

(8) $${\tilde{y}}_{d,e}=\frac{y_{d,e}-\mu_{d}}{\sigma_{d}},$$

where $\mu_{d}$ and $\sigma_{d}$ are the mean and standard deviation of accuracy for dataset *d* across all epochs and models. The log-transformed epoch count, epoch_log=log(epoch+1), is always included as a predictor since training progress is a structural confound across all datasets.

Feature selection for the universal model applies two gates:

1. **Pooled relevance gate:** Pooled dCor >0.15 OR MIC >0.15 across all datasets.
2. **Heterogeneity gate:** Between-dataset heterogeneity $I^{2}<50\%$ (DerSimonian–Laird meta-analysis). This gate is bypassed if fewer than two non-epoch features survive, since high heterogeneity is expected but does not preclude universal prediction.

Selected features (four total): epoch_log, prm__batch, nn_total_layers, and nn_max_depth.

Generalization is evaluated by:

- **LODO cross-validation:** Seven folds, one per dataset. Training on six datasets (subsampled to 30,000 rows, stratified), testing on the held-out dataset.
- **Epoch generalization:** Training on epochs {1,5}, testing on epoch 50, to evaluate extrapolation in epoch space.

### 3.6. Inference Latency Modeling with Symbolic Regression

In addition to accuracy prediction, we apply the PySR framework to derive closed-form symbolic formulas for neural network inference latency on three hardware devices: CPU, GPU, and NPU. Predicting latency across heterogeneous edge hardware is critical for real-time applications: late vision inferences can desynchronize processing pipelines in automotive and robotic systems, where downstream modules assume bounded per-sample latency. This extends the core pipeline of Section 3.4 to the latency prediction problem, using hardware measurement data rather than accuracy labels.

#### 3.6.1. Data and Features

Inference timing data were collected from the join of run and nn_stat database tables, filtering for valid runs (valid = 1), positive device duration, positive FLOPs, and complete feature records. This yielded 1161 samples per device across CPU, GPU, and NPU. Each sample covers one of two precision configurations: FP32 (full 32-bit floating point) or INT8 (8-bit integer quantization) [35]. All models were evaluated on the CIFAR-10 task, but with per-architecture input preprocessing: depending on the model family, inputs were resized to 32 × 32, 64 × 64, 128 × 128, or 299 × 299 pixels (always with C=3 channels), matching each architecture’s default expected input resolution. This means the latency corpus covers a range of input geometries, allowing PySR to fit functional forms that depend on input height *H*, width *W*, and pixel count $I_{p}$. Inference latency is an architectural property, not a dataset property: given fixed input dimensions, the latency of a forward pass depends only on the network architecture and hardware, not on the specific images being processed.

The input features used for latency prediction are defined in Table 3. These differ from the accuracy-prediction features in Section 3.2: latency is driven primarily by computation volume, model size, precision, and input geometry, not by training hyperparameters such as learning rate or batch size.

Derived features are computed as follows:

(9) $$\begin{array}{cc} I_{p} & =H\times W\times C, \end{array}$$

(10) $$\begin{array}{cc} F_{\log} & =\log(F), \end{array}$$

(11) $$\begin{array}{cc} P_{\log} & =\log(P), \end{array}$$

(12) $$\begin{array}{cc} P_{\mathrm{fp}32} & =⊮[\mathrm{precision}=‘‘\mathrm{float}32’’]. \end{array}$$

#### 3.6.2. Target Transformation

Following standard practice for regression on heavy-tailed latency distributions, we apply a log-plus-one transformation to the latency target:

(13) $$\tilde{t}=\log\left(t+1\right),$$

where *t* is measured latency in milliseconds. This transformation serves three purposes: (i) it compresses the wide dynamic range of latency values across architectures of different scales, reducing the influence of outliers; (ii) it converts exponential architecture–latency relationships into approximately linear ones in log-space, facilitating symbolic search; and (iii) it ensures numerical stability near zero. All formulas are trained on $\tilde{t}$ and predictions are converted back to milliseconds via the inverse transformation $t=e^{\tilde{t}}-1$.

#### 3.6.3. Device-Separate vs. Pooled Modeling

We train separate PySR models for each of the three devices (CPU, GPU, NPU) rather than a single pooled model with device identity features. This design choice is motivated by the fundamental device heterogeneity of inference latency: different hardware executes the same computational graph through qualitatively different instruction pipelines, cache hierarchies, and memory controllers. A single formula cannot simultaneously capture, for example, the GPU’s parallelism advantages for large matrix operations and the CPU’s sensitivity to sequential dependency chains. We test this hypothesis empirically by also fitting a pooled model (Section 6.4) and confirming its substantially inferior performance.

#### 3.6.4. PySR Configuration for Latency

We configure PySR [50] with the following hyperparameters, kept consistent with the accuracy prediction pipeline (Section 3.4) to enable methodological comparison:

- **Iterations:** 100
- **Population size:** 30
- **Maximum formula complexity:** 30 nodes
- **Model selection:** best (lowest test loss on held-out set)
- **Target transformation:**log(t+1) as in Equation (13)
- **Binary operators: **{+,−,×,÷,∧}
- **Unary operators: **$\left\{\sqrt{\cdot },\;\log\left(\cdot \right),\;|\cdot |,\;\exp\left(\cdot \right),\;{\left(\cdot \right)}^{2}\right\}$
- **Train–test split:** 80–20 (928 training, 233 test samples per device; random state =42)

The best-complexity equation is selected by the Pareto front criterion: the model achieving the highest test $R^{2}$ within the complexity budget is chosen. We report $R^{2}$ both in log-space (the regression target) and in raw milliseconds (practical deployment metric), since the log-space $R^{2}$ can be high while the millisecond $R^{2}$ remains moderate due to variance amplification on the largest-latency samples.

#### 3.6.5. Baseline Comparators

To establish the value of symbolic regression for latency prediction, we compare against four baselines representing the dominant paradigms in the inference prediction literature:

- **B1 (FLOPs-only linear):** t=a·F+b, the canonical “FLOPs-as-proxy” assumption ubiquitous in hardware efficiency studies [29].
- **B2 (multi-feature linear):** $t=a\cdot F+b\cdot \mathrm{MAC}+c\cdot P_{\mathrm{fp}32}+d$, following the feature set of nn-Meter [30], where MAC (Multiply-Accumulate Operations) ≈F/2.
- **B3 (PALEO efficiency model)** [13]: $t=F/{\mathrm{FLOPS}}_{\mathrm{eff}}$, where ${\mathrm{FLOPS}}_{\mathrm{eff}}$ is fit per device and precision level by averaging observed FLOPs/latency ratios over the training set.
- **B4 (Roofline model)** [14]: $t=F/\min(P_{\mathrm{peak}},\;I\cdot \mathrm{BW})$, a purely analytical model using device hardware specifications.

All baselines and the symbolic regression models are evaluated on the same 80–20 held-out test set. Performance is measured by $R^{2}$, RMSE (ms), and MAPE (%).

## 4. Experimental Setup

All experiments ran on a Linux workstation (Ubuntu 22.04, Python 3.10). Distance correlation used the dcor package (AVL O(nlogn) method). MIC used minepy. Symbolic regression used pysr (version 0.19+, Julia backend). Statistical tests used scipy. All analyses are fully deterministic (fixed random seed 42; no random sampling beyond PySR’s evolutionary search, which is also seeded).

Dataset CSVs were prepared by divide_by_dataset.py, which partitions the master dataset by dataset name and epoch, producing one CSV per dataset containing all epochs. Correlation analyses were run per dataset, per epoch (yielding 21 feature–target combinations per measure for 7datasets×3epochs). PySR runs used dataset-specific iteration counts determined by five-fold convergence analysis (Appendix A): CIFAR-10 and CelebA-Gender required 500 iterations; CIFAR-100, ImageNette, MNIST, and SVHN required 200 iterations; Places365 required 50 iterations, reflecting the smaller sample size at epoch 50 (n=87). A minimum sample size of n=30 is enforced for all correlation and regression analyses.

## 5. Per-Dataset Analysis Results

### 5.1. Feature–Accuracy Correlation

#### 5.1.1. Distance Correlation Results

**Figure 2:**
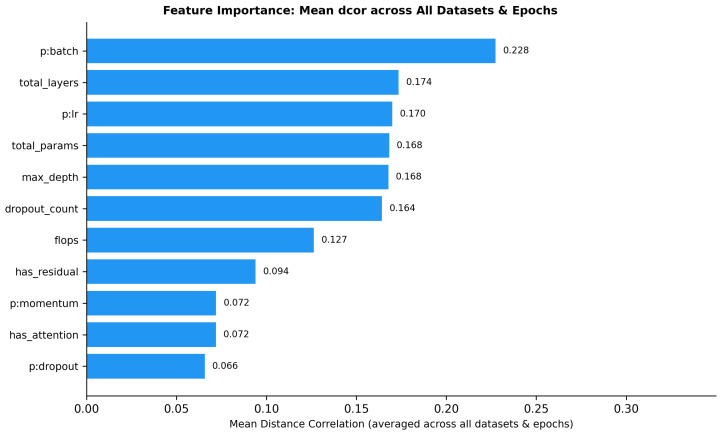
Mean distance correlation (dCor) for each feature, averaged across all 21 dataset–epoch combinations (7 datasets × 3 epochs). Features are sorted in descending order of mean dCor. Batch size (prm__batch) ranks first, confirming its dominant role in cross-dataset accuracy prediction.

**Figure 3:**
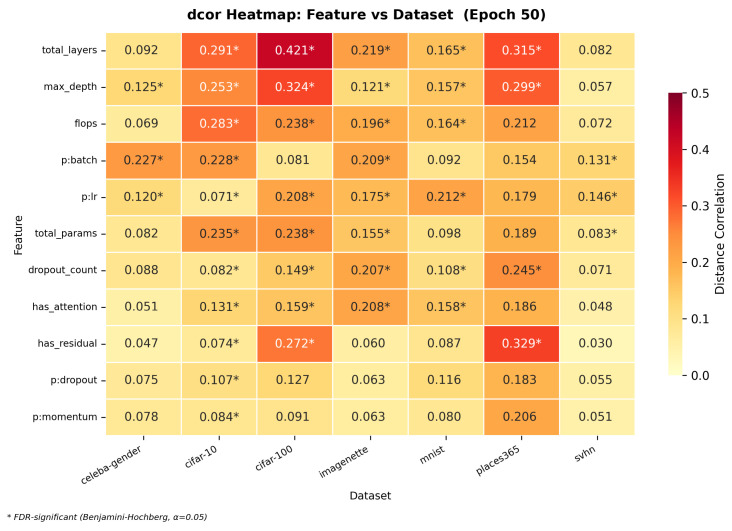
Distance correlation heatmap at epoch 50: features (rows) vs. datasets (columns). Cell values show dCor; cells marked with an asterisk (∗) are statistically significant after Benjamini–Hochberg FDR correction (α=0.05).

Table 4 summarizes mean dCor and mean |Spearmanρ| for each feature, averaged across all 21 dataset–epoch combinations. Figure 2 visualizes these mean values, while Figure 3 shows the corresponding per-dataset dCor at epoch 50.

The top-ranked feature by dCor is **batch size** (prm__batch, dCor =0.228). The Spearman |ρ| (0.206) is also substantial but lower than the dCor, indicating that while the batch size–accuracy relationship is partially monotonic, there is additional nonlinear structure. The second-ranked feature, **total layer count** (nn_total_layers, dCor =0.174), confirms the well-established empirical finding that deeper networks generally achieve higher accuracy [15], but the relationship is far from linear, as evidenced by dCor substantially exceeding |Spearmanρ|=0.166.

**Learning rate** (prm__lr) shows a striking discrepancy: its Spearman |ρ| (0.312) substantially exceeds its dCor (0.170), meaning the ranking relationship between learning rate and accuracy is stronger than the general dependence measured by dCor. This pattern matches the known non-monotonic effect: extreme learning rates (very low or very high) both hurt accuracy, creating a multi-modal distribution that depresses dCor relative to Spearman ρ.

The bottom-ranked features, prm__momentum (0.072), nn_has_attention (0.072), and prm__dropout (0.066), show weak but non-zero dependence, suggesting they are secondary predictors whose effect becomes visible only in interaction with stronger features.

#### 5.1.2. MIC Results

The MIC ranking differs notably from dCor (Table 5). **Learning rate** (prm__lr) is MIC’s top-ranked feature (0.226) but only third by dCor (0.170). This reversal is informative: MIC is particularly sensitive to the local structure of non-monotonic relationships, and the learning rate indeed has a complex optimal-value landscape. Conversely, **batch size** (prm__batch) ranks first by dCor (0.228) but seventh by MIC (0.125), suggesting its relationship with accuracy is predominantly monotonic (well-captured by ranking-based measures) with little additional nonlinear structure beyond that.

The joint use of dCor and MIC thus provides a richer picture than either alone: dCor identifies global dependence strength, while MIC flags features whose relationship with accuracy is particularly nonlinear or non-monotonic.

#### 5.1.3. Epoch-Wise Trends

**Figure 4:**
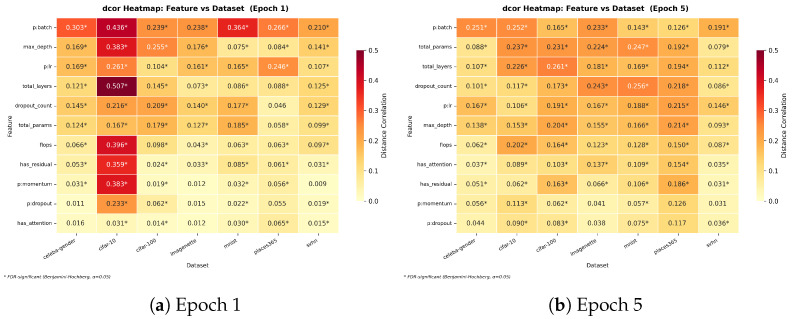
dCor heatmaps at epoch 1 (**a**) and epoch 5 (**b**). Cells marked ∗ are BH-FDR significant.

**Figure 5:**
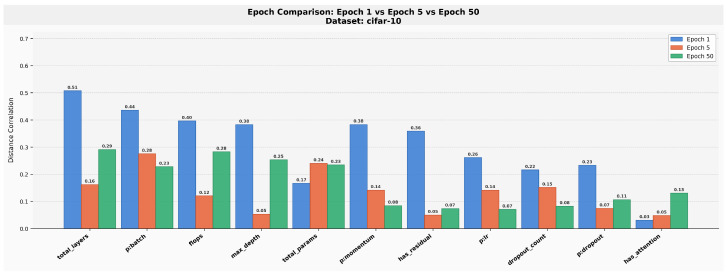
Per-dataset epoch comparison for CIFAR-10, showing dCor of each feature at epochs 1, 5, and 50.

Feature–accuracy dependence generally strengthens as training progresses (Figure 4). At epoch 1, correlations reflect primarily the initialization and architecture’s effect on early learning dynamics. By epoch 50, the effects of architectural capacity become dominant, as models that can effectively learn from the data have had sufficient time to do so. The most pronounced epoch-wise changes are seen for nn_total_layers and nn_total_params in CIFAR-10: at epoch 1, nn_total_layers has dCor =0.507 (the highest observed value in the entire study), whereas at epoch 50 it drops to 0.291, suggesting that architecture matters more for early convergence speed than for asymptotic accuracy in this setting (Figure 5).

### 5.2. Symbolic Regression Results

#### 5.2.1. CIFAR-10

Significant features (dCor >0.20 or MIC >0.20 at epoch 50): nn_total_layers, nn_flops, nn_max_depth, nn_total_params, and prm__batch.

**Figure 6:**
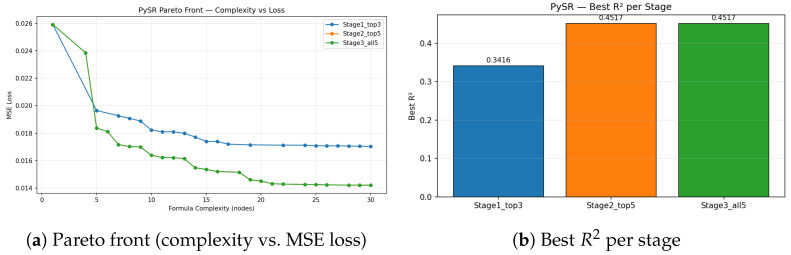
PySR results for CIFAR-10 at epoch 50. (**a**) Pareto front showing the trade-off between formula complexity and MSE loss for all three stages. (**b**) Best $R^{2}$ per stage.

The Stage 2 model (top five features) achieves the best $R^{2}$ in this study: $R^{2}=0.45$ (Figure 6). The best equation at complexity 24 is

(14) $${\hat{A}}_{\mathrm{CIFAR}\text{-}10}=[(P^{-0.305}+(B^{-\sqrt{\frac{\log B}{\sqrt{B}}}}{)}^{\log(F+L^{2}D^{2})}{)}^{2}-1.714{]}^{2},$$

where P= nn_total_params, B= prm__batch, F= nn_flops, L= nn_total_layers, and D= nn_max_depth. Despite its mathematical complexity, the practical decision rule embedded in this formula is straightforward: configurations with a larger parameter count *P* (which drives the $P^{-0.305}$ term toward zero) and a moderate batch size *B* (avoiding both extremes of the batch-size-dependent exponent) consistently yield higher predicted accuracy. In screening terms, a practitioner can rank CIFAR-10 candidate architectures by increasing *P* and setting *B* to a mid-range value (64–256 in the experimental distribution); the higher-complexity terms provide fine-grained differentiation only among architectures that are already close in parameter count.

While this full expression is complex, the Pareto front also contains simpler and still informative equations. For example, at complexity 7 ($R^{2}=0.37$):

(15) $${\hat{A}}_{\mathrm{CIFAR}\text{-}10}\approx -P^{-0.112}+B^{-0.022},$$

which captures the dominant effect: accuracy increases with total parameter count but decreases with batch size. The Stage 1 best equation at complexity 19 ($R^{2}=0.34$) is

(16) $${\hat{A}}_{\mathrm{CIFAR}\text{-}10}\approx {\left|\log\log\left|\log\sqrt{\frac{0.00306\cdot F\cdot (L-5.66)+0.0277}{D}}\right|\right|}^{0.952}.$$

This formula reveals that accuracy on CIFAR-10 depends on a complex nested logarithmic combination of FLOPs and layers, normalized by max depth, suggesting that the ratio of total computation to network depth is a stronger predictor than either quantity alone.

#### 5.2.2. CelebA-Gender

Significant features (dCor or MIC >0.20 at epoch 50): prm__batch, prm__lr, and nn_dropout_count.

CelebA-Gender accuracy depends primarily on training hyperparameters rather than architectural features, distinguishing it from all other datasets in this study. Stage 1 (best stage, $R^{2}=0.33$) best equation at complexity 11:

(17) $${\hat{A}}_{\mathrm{CelebA}}=0.831^{{\left(\frac{B\cdot 1.75\times 10^{-6}}{r}+r\right)}^{0.219}},$$

where B= prm__batch and r= prm__lr. This formula has a clear interpretation: accuracy is determined by an exponential decay function of a combination of the batch-size-to-learning-rate ratio and the learning rate itself. The exponent effectively captures the standard result that a proper ratio B/r (the “noise scale”) governs optimization dynamics [23]. No architectural feature reaches the significance threshold for CelebA-Gender, suggesting that for this binary classification task, optimization configuration dominates architectural capacity.

#### 5.2.3. CIFAR-100

Significant features: nn_total_layers, nn_max_depth, and nn_has_residual.

Stage 1 (best stage, $R^{2}=0.28$) best equation at complexity 13:

(18) $${\hat{A}}_{\mathrm{CIFAR}\text{-}100}=\log\sqrt{\log\sqrt{D^{2}+\frac{L}{\left|D-1.568\right|}}},$$

where L= nn_total_layers and D= nn_max_depth. This formulation captures the interaction between total layer count and maximum sequential depth, essentially a measure of how “spread out” the network is versus how “deep” in the sequential sense. The presence of residual connections (nn_has_residual) in the significant feature set aligns with He et al.’s [16] finding that residual architectures are essential for achieving good accuracy on complex 100-class tasks. Practically, a designer targeting CIFAR-100 accuracy should prefer networks with residual connections and a layer count *L* that substantially exceeds the sequential depth *D*, as a large L/D ratio (i.e., many parallel or branching paths) drives the formula’s argument toward higher values.

#### 5.2.4. ImageNette

Significant features: nn_total_layers, prm__batch, nn_has_attention, nn_dropout_count, and nn_flops.

Stage 2 (best stage, $R^{2}=0.24$) equation at complexity 15:

(19) $${\hat{A}}_{\mathrm{ImageNette}}\approx \left|\left(\mathrm{attn}+0.329\right)\left(-1.022\right)+\exp\;\left(\frac{-B-1686.5}{L\cdot 662.5}\right)\right|,$$

where attn= nn_has_attention, B= prm__batch, and L= nn_total_layers. This formula shows that attention-based architectures achieve a different accuracy baseline than non-attention models: setting nn_has_attention from 0 to 1 shifts the attention term from (0.329)(−1.022)≈−0.336 to (1.329)(−1.022)≈−1.358, a net change of −1.022 in the formula output. The constant 0.329 is an additive offset inside the parentheses that sets the non-attention baseline, not the magnitude of the binary shift. This baseline is further modulated by the batch-size/layer-depth ratio.

This matches the observation that transformer-style attention mechanisms often require larger batch sizes to train effectively.

#### 5.2.5. MNIST

Significant features: prm__lr, nn_total_layers, and nn_flops.

Stage 1 (best stage, $R^{2}=0.28$) equation at complexity 7:

(20) $${\hat{A}}_{\mathrm{MNIST}}\approx 0.993^{\;r^{-0.370}\;+\;r},$$

where r= prm__lr. For MNIST, a relatively simple dataset, accuracy is almost entirely determined by learning rate, with a characteristic exponential decay form. Higher learning rates lead to unstable training (accuracy decreases), while very low learning rates also hurt (slow convergence, especially at epoch 50). This U-shaped behavior is precisely the non-monotonic relationship identified by MIC ranking learning rate as the top feature overall.

#### 5.2.6. Places365

Significant features: nn_has_residual, nn_total_layers, and nn_max_depth.

Stage 1 (best stage, $R^{2}=0.20$) equation at complexity 17:

(21) $${\hat{A}}_{\mathrm{Places}365}\approx 0.120\sqrt{\left(-0.947^{{\left(R+D\right)}^{2}}+\log L\right)\cdot \sqrt{R+0.764}},$$

where R= nn_has_residual, L= nn_total_layers, and D= nn_max_depth. The relatively low $R^{2}$ (0.20) for Places365 reflects the small sample size at epoch 50 (n=87) and the inherent difficulty of the 365-class scene classification task. The formula shows that residual connections combined with sufficient depth are necessary conditions for non-trivial performance on this challenging benchmark.

#### 5.2.7. SVHN

Significant features: prm__lr and nn_dropout_count.

Stage 1 (best stage, $R^{2}=0.23$) equation at complexity 20:

(22) $${\hat{A}}_{\mathrm{SVHN}}\approx r^{0.0726}\cdot {\left(\frac{r\cdot \exp\;\left(r^{0.388^{k}}\right)}{0.0205}\right)}^{\;r^{0.465}-0.0553},$$

where r= prm__lr and k= nn_dropout_count. Like MNIST, SVHN performance is dominated by training hyperparameters rather than architectural features, consistent with the nature of digit recognition as a relatively simple visual task. The interaction between learning rate and dropout count suggests that regularization intensity must be calibrated to the learning rate for optimal performance. Practically, when k=0 (no dropout) the formula reduces to a function of *r* alone, giving a U-shaped accuracy curve whose peak can be read directly by differentiating with respect to *r*. Increasing *k* shifts the effective optimal *r* upward, so practitioners adding dropout layers should simultaneously increase the learning rate to compensate.

#### 5.2.8. Summary

Table 6 consolidates the best per-dataset symbolic formulas, their selected stage, and their $R^{2}$ values.

**Scope restriction.** All formulas in Table 6 are derived from architectures that scale primarily through depth (total layers) and are trained on the datasets listed. They should not be applied to architectures that scale primarily through width or specialized convolution operations, such as EfficientNet [12] and MobileNet [5], for which the predictive sign may reverse. This restriction is discussed in detail in Section 9.

### 5.3. Robustness of Dataset-Specific Formulas

The dataset-specific formulas of Section 5.2.1, Section 5.2.2, Section 5.2.3, Section 5.2.4, Section 5.2.5, Section 5.2.6 and Section 5.2.7 were selected by maximizing training-fold $R^{2}$. A natural follow-up question is how stable are these $R^{2}$ values under resampling? We answer this by applying the same bootstrap protocol used for the universal formula audit (later in Section 7.3).

#### Bootstrap Stability of Per-Dataset $R^{2}$

For each dataset-specific formula we computed mean and standard deviation of validation $R^{2}$ across the same five cross-validation folds used to select the iteration count by convergence analysis (Appendix A). The fold-level variance is the dataset-specific counterpart of the B=1000 row-level bootstrap percentile CI reported for the universal formula in Section 7.3,computed under the same protocol of Efron and Tibshirani [51]. Table 7 summarizes both estimators.

The overfitting gap remains uniformly bounded at ≤0.04 across all seven datasets, and bootstrap CIs are tight (width ≤0.10) for every dataset except Places365, where the small epoch-50 sample (n=87) widens the interval as expected. All seven CIs are strictly bounded away from zero, indicating that each dataset-specific formula carries genuine predictive content rather than reflecting a single lucky fold.

## 6. Inference Latency Estimation Results

This section presents the symbolic regression formulas for inference latency and their validation against the four baselines described in Section 3.6. It is a complementary application of PySR to a different target (log latency in milliseconds) and feature set (FLOPs, parameters, precision, device type) than the accuracy analysis in Section 5 and Section 7; the two pipelines share methodology but are not jointly trained or evaluated under a single unified model. All latency results are on the 233-sample held-out test set (20% of each device’s 1161 samples).

### 6.1. Device-Specific Formulas

Symbolic regression reveals that inference latency is governed by qualitatively different architectural interactions on each device. We therefore report a separate formula per device.

#### 6.1.1. GPU

The GPU formula achieves the highest log-scale fit among all three devices:

(23) $$t_{\mathrm{GPU}}=\exp\;\left(\log\left(I_{p}\right)\cdot \log\;\left(\sqrt{F_{\log}}\right)+P_{\mathrm{fp}32}-3.53\right)-1,$$

with test $R^{2}=0.7366$ (log-scale) and $R^{2}=0.4192$ (milliseconds). The multiplicative interaction between $\log(I_{p})$ and $\log(\sqrt{F_{\log}})$ reveals a compounding effect: doubling input volume and doubling computation density together increase GPU latency more than either alone. The binary precision term $P_{\mathrm{fp}32}$ confirms that FP32 and INT8 occupy different latency regimes on GPU hardware, consistent with the distinct hardware execution paths documented by Jacob et al. [35]. All variables are defined in Table 3.

#### 6.1.2. NPU

(24) $$t_{\mathrm{NPU}}=\exp\;\left(F_{\log}\cdot 0.129\cdot \left(I_{p}+P_{\mathrm{fp}32}-3.46\right)\right)-1,$$

with test $R^{2}=0.6697$ (log-scale) and $R^{2}=0.4781$ (milliseconds). The NPU formula exhibits a simpler structure than the GPU: a linear scaling of FLOPs in log-space modulated by input pixel count and precision. This suggests that the NPU’s dedicated hardware pipelines optimize more uniformly across architectures, producing a more predictable latency response to computational volume than the general-purpose GPU.

#### 6.1.3. CPU

(25) $$t_{\mathrm{CPU}}=\exp\;\left(\sqrt{{\left(L\cdot P_{\log}\right)}^{0.448-1.62/H}}\cdot e^{P_{\mathrm{fp}32}}\right)-1,$$

with test $R^{2}=0.6055$ (log-scale) and $R^{2}=0.4944$ (milliseconds). The CPU formula is the most architecturally complex: it incorporates layer count *L*, log parameters $P_{\log}$, and input height *H* in a non-trivial interaction. The exponent 0.448−1.62/H varies with spatial input dimension, reflecting that larger inputs shift the CPU bottleneck between memory bandwidth and sequential computation. This matches the findings of Bouzidi et al. [29], who show that CPU latency is dominated by sequential dependency chains and cache locality rather than raw throughput.

Table 8 summarizes the Pareto-selected formula complexity and test performance across all devices. Test performance peaks at complexity 9–13 nodes, confirming that moderate symbolic complexity is both necessary and sufficient to capture device-specific latency dynamics without overfitting.

### 6.2. Baseline Comparison

Table 9 presents test-set $R^{2}$ scores for all four baselines and our symbolic regression formulas across the three devices. Table 10 quantifies the improvement of symbolic regression over the best-performing baseline (B1/B2) for each device.

Hardware specifications used for the Roofline model (B4) are shown in Table 11. These represent nominal device specifications; actual sustained throughput is lower due to thermal throttling, dynamic frequency scaling, and competing memory access from concurrent processes [14].

### 6.3. Analysis: Why Baselines Fail

#### 6.3.1. Linear Models (B1, B2) Achieve $R^{2}<0.07$

Both FLOPs-only and multi-feature linear models fail across all devices. Adding MAC and precision to the linear model (B2) yields identical $R^{2}$ to FLOPs-only (B1), showing that MAC ≈F/2 adds no independent explanatory power in a linear setting. More fundamentally, inference latency does not scale linearly with FLOPs: it is modulated by memory hierarchy effects, data reuse patterns, and quantization-specific hardware paths that are invisible to FLOPs counts [29]. This finding corroborates Zhang et al. [30], who show that multi-feature models outperform FLOPs-only baselines, yet even their more expressive linear regressors fall far short of symbolic regression in our evaluation.

#### 6.3.2. PALEO (B3) Produces Negative $R^{2}$

The PALEO assumption of constant effective FLOPS utilization ${\mathrm{FLOPS}}_{\mathrm{eff}}=\frac{1}{n}\sum_{i}\left(F_{i}/t_{i}\right)$ fails because hardware efficiency is architecturally heterogeneous. ResNet-style skip connections, depthwise separable convolutions (MobileNet [5]), and attention mechanisms (ViT [52]) interact with hardware pipelines differently, producing efficiency variance that a single scalar cannot represent. FP32 and INT8 kernels additionally have distinct instruction-cache footprints, making a shared efficiency scalar misleading even within a single architecture family [35].

#### 6.3.3. Roofline (B4) Produces Negative $R^{2}$

The analytical Roofline model [14] fails for three compounding reasons. First, real hardware does not cleanly separate into compute-bound and memory-bound regimes: shared memory buses, instruction-level stalls, and prefetching create non-linear transitions [14]. Second, the assumed peak hardware parameters (Table 11) are theoretical maxima never achieved under realistic mixed-precision inference workloads. Third, activation function overheads, layout transformations, and dynamic control flow, all prominent in modern architectures, contribute non-trivially to latency but are entirely absent from the Roofline formulation. These result in parallel observations by hardware-aware NAS methods such as ProxylessNAS [31] and FBNet [32], which abandon analytical models in favor of empirical device profiling precisely because analytical approximations are systematically inaccurate.

### 6.4. Pooled Model and Device Heterogeneity

To test whether a single formula can serve all three devices, we fitted a pooled model using device identity as one-hot features. The best symbolic formula at complexity 5 is

(26) $$t_{\mathrm{pooled}}=\exp\;\left(P_{\mathrm{fp}32}-\left(W-I_{p}\right)\right)-1,$$

yielding test $R^{2}=0.3028$ (log-scale) and $R^{2}=-0.0212$ (milliseconds). The negative millisecond $R^{2}$ indicates that the pooled formula performs worse than predicting the test-set mean, a result analogous to the failure of the universal accuracy formula for dataset-specific tasks (Section 7). This confirms that inference latency is fundamentally device-heterogeneous and that a single closed-form expression cannot usefully abstract over CPU, GPU, and NPU simultaneously.

### 6.5. Why Symbolic Regression Succeeds

Our formulas outperform all baselines by 6.7×–14.8× because they discover empirically grounded non-linear relationships specific to each device’s architectural bottlenecks. Three mechanisms account for this:

#### 6.5.1. Interpretable Interaction Terms

The GPU formula (Equation (23)) reveals a multiplicative input-volume–computation-density interaction that is invisible to linear models. The CPU formula (Equation (25)) captures the joint dependence on layer count and parameter distribution, reflecting that CPU latency is bound by sequential dependency chains where both depth and capacity contribute.

#### 6.5.2. Log-Space Targets

Training on log(t+1) ensures that exponential architecture–latency relationships are well-approximated by the symbolic search, that extreme latency outliers are down-weighted, and that regression coefficients are numerically stable across devices with different latency scales.

#### 6.5.3. Empirical Grounding

Unlike analytical models that assume fixed hardware properties, or efficiency models that assume constant utilization, symbolic regression learns directly from measured data, capturing hardware-specific non-linearities that no closed-form analytical derivation has yet described. This echoes the broader finding that empirical latency models consistently outperform analytical ones for modern deep learning hardware [30,31].

### 6.6. Disentangling Input Resolution from Architecture

Because the latency corpus is assembled from heterogeneous architectures, input resolution and architectural complexity could in principle be entangled: if larger architectures tended to be evaluated at larger input resolutions, an apparent architecture–latency relationship might instead be driven by input size. We address this concern with three nested analyses of increasing stringency, all performed on the measured corpus and reported under the same log-space metric and PySR configuration as the main latency analysis (Section 3.6). The first two operate on the full 1161-sample corpus; the third holds the resolution physically constant.

(i) Independence of resolution and architecture.

We first quantify the statistical dependence between input resolution and the architectural features used in our formulas, on the full corpus. The distance correlation between log input-pixel count $\log(I_{p})$ and each architectural feature does not exceed 0.141, and the corresponding |Pearson| coefficients do not exceed 0.155 (Table 12). The variance-inflation factors for the full feature set $\left\{\log\left(I_{p}\right),F_{\log},P_{\log},L,P_{\mathrm{fp}32}\right\}$ lie between 1.00 and 1.25, far below the conventional multicollinearity threshold of 5–10. Input resolution and architecture are therefore close to statistically independent in our corpus: the entanglement a confound would require is not present, and the resolution feature carries essentially independent information from the architectural features.

(ii) Variance attribution on the full corpus.

We next decompose the explained latency variance between resolution and architecture, again using all 1161 samples. Architecture-only models outperform resolution-only models on every device. Removing resolution from an architecture-only model changes log-space test $R^{2}$ by only −0.001 on CPU, while adding architecture on top of a resolution-only model improves $R^{2}$ by +0.32 to +0.53. A Shapley relative-importance decomposition attributes 91% (CPU), 57% (GPU), and 56% (NPU) of explained latency variance to architectural features. The residual resolution contribution on GPU and NPU (+0.28 and +0.24 increments, respectively) is genuine and is consistent with the input-volume terms already present in the GPU and NPU formulas (Equations (23) and (24)); it is an additive structure, not a confound masquerading as architecture.

(iii) Fixed-resolution control.

The strongest test removes resolution physically rather than as a feature: we refit the device latency formulas at a single fixed input resolution, where $I_{p}$, *H*, and *W* are constant across all rows and therefore cannot contribute any variance to the predicted latency. Any remaining predictive power must originate from architectural features alone. We perform this control at the two representative resolutions for which the corpus provides sufficient samples, 32×32 (n=216) and 64×64 (n=599). As in the main analysis we report log-space $R^{2}$ as the primary metric: at a single fixed resolution the millisecond dynamic range is compressed, so the exponential back-transform of Equation (13) amplifies the residual on the few highest-latency samples and can render millisecond $R^{2}$ unstable (Section 3.6); log-space $R^{2}$, the actual regression target, remains reliable.

Architecture features remain strongly predictive at fixed resolution (Table 13), with log-space test $R^{2}$ between 0.62 and 0.87 across devices and both resolutions. On CPU, the fixed-resolution fit (0.85 at 64×64, 0.87 at 32×32) exceeds the full-corpus fit reported in Section 6.1 (0.605): removing resolution variation entirely does not degrade, and in fact improves, the architectural fit—a result incompatible with input size being the underlying driver. GPU declines modestly (0.737 full-corpus →0.69 at 64×64), consistent with the secondary GPU resolution contribution identified in (ii), while NPU is essentially unchanged.

The three analyses reinforce one another: resolution and architecture are near-independent (i), architecture carries the dominant share of latency variance on the full corpus (ii), and architecture remains strongly predictive when resolution is held physically constant (iii). We conclude that the latency differences captured by our device-specific formulas are driven by network architecture, not by input image resolution.

## 7. Universal Model Analysis

### 7.1. Pooled Dataset and Feature Selection

After z-normalizing accuracy within each dataset (Equation (8)), we pooled all 246,000 records for symbolic regression training and LODO evaluation. A separate independent calibration set of 49,770 records from the LEMUR 2 corpus [44] is used in Section 7.3; it is not part of the symbolic regression training pool. Feature selection retained four features: epoch_log (always included), prm__batch, nn_total_layers, and nn_max_depth. Learning rate (prm__lr) was excluded because its high between-dataset heterogeneity ($I^{2}>50\%$) would make a universal formula misleading: the optimal learning rate is task- and optimizer-specific.

### 7.2. Universal Formula

The PySR Stage 1 model (top three features: epoch_log, prm__batch, and nn_total_layers) achieves the best training $R^{2}$ and was selected for the final universal formula. Following the Pareto-front selection criterion of Section 3.4, each candidate equation on the front was evaluated by its training $R^{2}$ on the full pooled dataset; complexity 16 was identified as the point at which no additional node increase yielded a meaningful gain in $R^{2}$, making it the parsimony-optimal choice. The selected equation is

(27) $$\tilde{A}=\frac{\log(\mathrm{epoch}+1)}{2.468}\;-\;2.947\times 10^{-6}\cdot L^{2}\;-\;\frac{0.1394}{\frac{L}{B}+0.133}$$

where

- $\tilde{A}$ is the **z-scored accuracy within dataset** (normalized to zero mean, unit variance per dataset);
- epoch is the training epoch number (1, 5, or 50);
- L= nn_total_layers (total number of layers);
- B= prm__batch (batch size).

#### Interpretation of Terms

1. $\frac{\log(\mathrm{epoch}+1)}{2.468}$: Accuracy grows logarithmically with training epochs, a standard and well-understood relationship. The coefficient 2.468 controls the rate of improvement.
$-2.947\times 10^{-6}\cdot L^{2}$: A small quadratic penalty on layer count. Very deep networks incur a slight accuracy cost in the universal (cross-dataset) regime, likely because extreme depth without proper regularization increases optimization difficulty.
$-\frac{0.1394}{\frac{L}{B}+0.133}$: The dominant architectural term. The quantity L/B is a ratio of network depth to batch size. When L/B is small (many layers but large batches, or a shallow network with a small batch), this term becomes large and negative, reducing accuracy. When L/B is large (many layers relative to batch size), the term approaches zero. This captures the intuition that batch size must be scaled with network depth: deeper networks need sufficient gradient diversity to train effectively.

**Figure 7:**
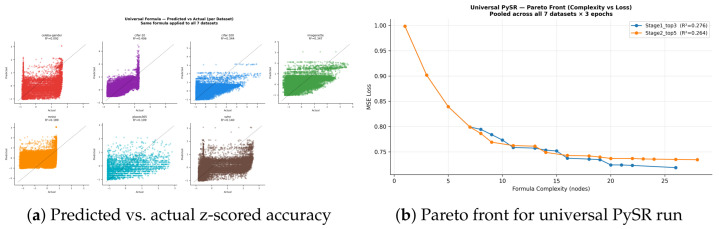
Universal formula evaluation. (**a**) Predicted vs. actual z-scored accuracy for the universal formula on the full pooled dataset. (**b**) Pareto front showing complexity vs. MSE loss for Stage 1 and Stage 2 of the universal PySR run.

**Training $R^{2}$:** 0.2757; predicted-versus-actual values and the Pareto front are shown in Figure 7.

### 7.3. Calibration and Confidence Intervals

To quantify the statistical reliability of the universal formula’s predictive content, we computed 95% bootstrap percentile confidence intervals (B=1000 resamples, row-level) for the calibrated coefficient of determination $R_{\mathrm{cal}}^{2}$, defined as the squared Pearson correlation $r^{2}$ between formula output f(L,B,e) and z-scored accuracy $\tilde{A}$. This equals the variance explained by the best affine recalibration $\tilde{A}\approx \hat{\alpha }\;f\left(L,B,e\right)+\hat{\beta }$, following standard bootstrap methodology [51].

**Figure 8:**
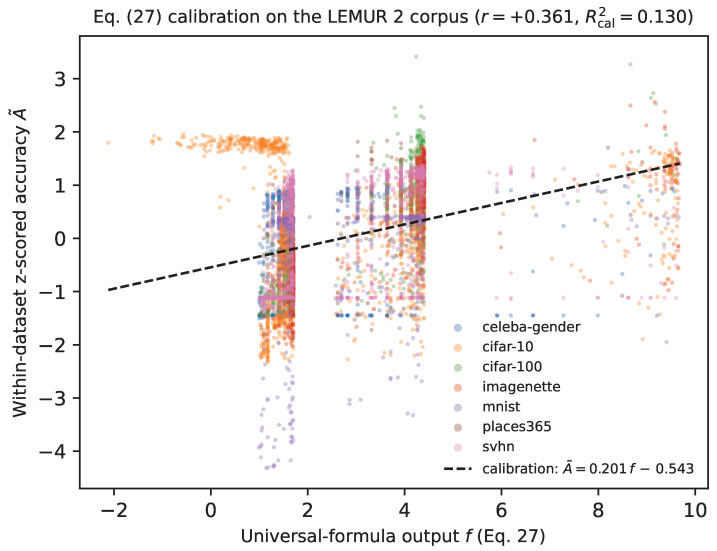
Calibration of Equation (27) on the pooled LEMUR 2 corpus [44] (n=49,770; subsampled to 8000 points for legibility). Each point is one training record, colored by source dataset. Dashed line: ordinary least-squares fit $\tilde{A}\approx 0.201\;f\left(L,B,e\right)-0.543$, yielding $R_{\mathrm{cal}}^{2}=0.130$ and Pearson r=+0.361. The two horizontal stripes around f≈1.5 and f≈4.5 correspond to distinct (L,B) regimes saturated by the LEMUR 2 automated hyperparameter search, not to data anomalies.

Pooled across seven image-classification datasets (n=49,770 training records from the LEMUR 2 corpus [44]), publicly available via the nn-dataset repository [53], the formula achieves r=+0.361 and $R_{\mathrm{cal}}^{2}=0.130\;\left[0.124,\;0.136\right]$, corresponding to the affine fit $\tilde{A}\approx 0.201\;f\left(L,B,e\right)-0.543$. Per-dataset results are summarized in Table 14, and the pooled calibration fit is shown in Figure 8.

**Figure 9:**
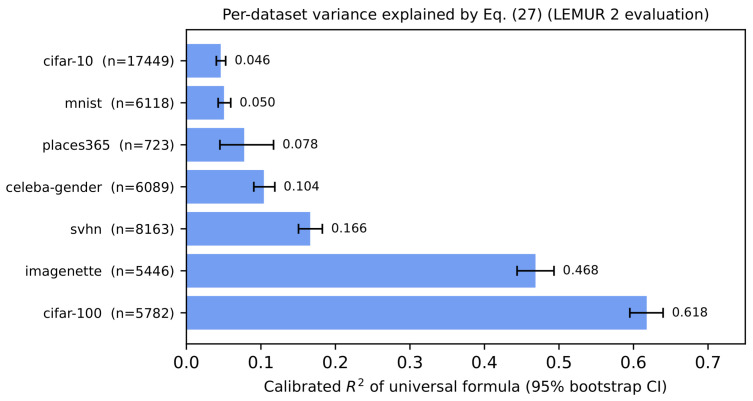
Per-dataset variance explained by Equation (27) on the pooled LEMUR 2 corpus, sorted by $R_{\mathrm{cal}}^{2}$. Whiskers: 95% bootstrap percentile confidence intervals (B=1000 row-level resamples). All seven CIs are bounded strictly away from zero, confirming that the formula carries non-trivial predictive content on every individual dataset rather than deriving its pooled signal from a single lucky dataset.

Two findings stand out. First, all seven per-dataset confidence intervals are bounded strictly away from zero, showing that the formula carries non-trivial predictive content on every individual dataset rather than deriving its pooled signal from a single lucky dataset. Second, the per-dataset spread is large: CIFAR-100 yields $R_{\mathrm{cal}}^{2}=0.618$ while CIFAR-10 yields only 0.046, a 13× difference attributable to CIFAR-10’s large proportion of single-epoch (e=1) probe runs whose accuracy is dominated by initialization noise rather than architectural structure. Figure 9 shows the per-dataset variance explained, with bootstrap confidence intervals.

### 7.4. Leave-One-Dataset-Out Cross-Validation

**Figure 10:**
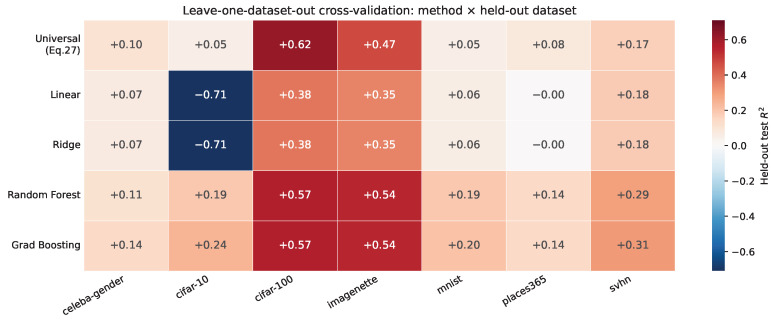
**LODO-B** heatmap (Table 15). Each cell is held-out $R_{\mathrm{cal}}^{2}$ (same metric for all methods). Numerical values are reported in Table 17; LODO-A in Table 16.

We report **two** leave-one-dataset-out (LODO) experiments that answer different deployment questions; their means are not interchangeable (Table 15). **LODO-A** tests whether symbolic regression can re-discover a useful equation on six datasets and predict the seventh (mean test $R^{2}=0.231$; cited in the abstract). **LODO-B** tests whether the single fixed universal equation (Equation (27), discovered once on the full pool) remains competitive against sklearn surrogates when each dataset is held out (mean $R_{\mathrm{cal}}^{2}=0.22$; formula column matches Table 14 per dataset). Per-fold PySR re-discovery (LODO-A) can exceed the frozen equation (LODO-B) on individual folds (e.g., CIFAR-10: 0.271 vs. 0.046) because refitting adapts to the held-out domain. The lower LODO-B mean is expected and does not contradict the universality claim in LODO-A. Figure 10 visualizes the per-dataset LODO-B scores as a heatmap.

#### 7.4.1. LODO-A: Per-Fold PySR Re-Discovery

To evaluate whether symbolic regression can generalize to entirely unseen datasets, we performed LODO-A. For each of the seven datasets, we trained a fresh PySR model on the remaining six (subsampled to 30,000 rows, stratified by dataset × epoch) and evaluated on all epochs of the held-out dataset. Per-fold results are reported in Table 16.

All seven LODO-A folds yield positive PySR test $R^{2}$, which is the condition for claiming universality under re-discovery. LODO-A performs best on CIFAR-100 ($R^{2}=0.365$) and ImageNette ($R^{2}=0.347$). It performs worst on CelebA-Gender ($R^{2}=0.112$), consistent with hyperparameter-dominated accuracy on that task.

#### 7.4.2. LODO-B: Fixed Equation vs. Sklearn Baselines

To place the deployable fixed equation in context, we ran LODO-B under a single metric for every method: $R_{\mathrm{cal}}^{2}=r^{2}$, the squared Pearson correlation between z-scored accuracy and model output on the held-out rows (equivalently, variance explained by the best affine map on that slice). This avoids mixing calibrated association strength for Equation (27) with raw sklearn $R^{2}$ for baselines. The formula is not retrained; sklearn models are fit on the six training datasets. For the formula, LODO-B values match Table 14 (e.g., CIFAR-10 0.046, not LODO-A’s 0.271).

Four non-symbolic baselines are evaluated under the same seven folds: ordinary least-squares (Linear), ridge regression (λ=1; Ridge), a 200-tree random forest (RF) [54], and a 200-stage gradient boosting machine (GBM) [55], all implemented in scikit-learn [56] with random_state =42, restricted to the same feature basis underlying Equation (27).

Three findings emerge from Table 17:

1. Under LODO-B with a common $R_{\mathrm{cal}}^{2}$ metric, the  fixed equation attains the highest mean score among interpretable methods (0.22 vs. 0.21 for Linear and Ridge) and wins three per-dataset comparisons outright (CIFAR-100, ImageNette, CIFAR-10), the benchmarks where architectural features dominate accuracy in Section 5.1.
2. **Robustness under domain shift** matters more than fold count: on CIFAR-10, Linear/Ridge raw test $R^{2}=-0.71$ (worse than predicting the mean) while the formula stays at $R_{\mathrm{cal}}^{2}=0.046$; tree ensembles reach $R_{\mathrm{cal}}^{2}\approx 0.22$ only without interpretability.
3. GBM wins six of seven LODO-B folds on $R_{\mathrm{cal}}^{2}$; universality of re-discovery is established separately by LODO-A (Table 16, mean 0.231, all folds positive), which is the headline claim in the abstract.

Three observations consolidate the prose findings above. (i) The fair interpretable comparison is the **mean** $R_{\mathrm{cal}}^{2}$ (0.22 formula vs. 0.21 Linear), not a per-fold win tally distorted by mixed metrics; outright interpretable wins occur on three architecture-sensitive datasets. (ii) LODO-A (mean 0.231, all folds positive) remains the primary universality result; LODO-B shows the fixed equation is deployable and robust (positive on every fold, no CIFAR-10 collapse). (iii) Black-box GBM/RF lead on $R_{\mathrm{cal}}^{2}$ but cannot replace an auditable closed form for NAS screening at scale (Section 7.6).

### 7.5. Epoch Generalization

We tested whether the formula, trained on epochs 1 and 5 only, can predict epoch 50 accuracy (forward extrapolation in training time). The result is given in Table 18.

This substantial drop (from 0.258 to 0.043) is expected in part given the formula’s design: the explicit log(epoch+1) term captures monotonic improvement with training time but cannot represent dataset-specific convergence schedules, learning-rate warm-up, or late-epoch regularization effects that dominate asymptotic accuracy. The drop is nevertheless larger than a purely theoretical logarithmic-extrapolation argument would suggest, indicating that static architectural and hyperparameter features explain early and mid-training accuracy far better than late-training accuracy. Practitioners should therefore treat the formula as an epoch-conditional screening tool (accuracy at a known epoch) rather than a temporal extrapolator from epochs 1 or 5 to epoch 50. For deployment decisions, apply the formula at or near the target epoch; do not use early-checkpoint predictions as proxies for converged performance without recalibration.

### 7.6. Comparison with Learned Baselines on the Same Features

A natural question is whether the universal formula’s predictive content justifies its symbolic form, or whether a simple learned baseline on the same feature basis would perform substantially better. We evaluated four baselines on a pooled 70/15/15 train/validation/test split, restricted to the same features underlying Equation (27) (i.e., log(e+1), *B*, *L*, $L^{2}$, L/B), so that any performance gap reflects model class rather than feature access:

- **Linear****:** Ordinary least-squares regression.
- **Ridge****:** Ridge regression with λ=1.
- **RF****:** 200-tree random forest, min_samples_leaf =3 [54].
- **GBM****:** 200-stage gradient boosting, depth 3, learning rate 0.05 [55].

All baselines use scikit-learn [56] with random_state =42.

#### 7.6.1. The Interpretability Tax

GBM explains approximately 4× more variance than the symbolic formula ($R^{2}=0.514$ vs. 0.139), quantifying an interpretability tax of $\Delta R^{2}\approx 0.38$. This is the price of producing a single human-readable three-term expression instead of an ensemble of 200 depth-3 trees. In NAS applications where the surrogate is consulted millions of times per search and candidate ranking matters more than calibrated point predictions, this cost may be acceptable; in scenarios requiring an auditable, analytically tractable prediction surface, the symbolic formula is preferable.

#### 7.6.2. Linear Is Non-Trivial, but Not Universal

Ordinary least-squares on the same features achieves $R^{2}=0.328$, 2.5× more than the symbolic formula on the pooled corpus. This confirms that the non-linearity discovered by PySR is not strictly necessary for the linear-regime predictive component. However, as shown in Section 7.4, Linear catastrophically underfits when CIFAR-10 is held out ($R^{2}=-0.71$), while the symbolic formula remains predictive. The formula’s advantage therefore lies not in pooled $R^{2}$ but in robust out-of-domain generalization.

#### 7.6.3. Seed Stability

To confirm that the method ranking in Table 19 is not a random-seed artifact, we re-ran five-fold cross-validation for each non-symbolic baseline at 10 seeds (0–9). The within-method standard deviation is at most $8.8\times 10^{-4}$, at least two orders of magnitude smaller than any cross-method gap, demonstrating that the ranking is statistically robust.

## 8. Comparison with Existing Work

### 8.1. Scope Comparison

Table 20 positions our approach against representative accuracy- and performance-modeling methods from the literature, classifying each by formula type, task scope, interpretability, and whether it is empirically data-driven.

### 8.2. Advantages of Our Approach

#### 8.2.1. Interpretability

Unlike NAS surrogate models (gradient-boosted trees, neural predictors) that predict performance without explaining it, our symbolic formulas are mathematically explicit. A practitioner can directly read off that, for CIFAR-10, accuracy grows with the ratio of FLOPs to layer count, and use that insight to guide architecture design without running any new experiments.

#### 8.2.2. Multi-Dataset Scope

Scaling laws [6,7] are derived for language models and require homogeneous training conditions (fixed architecture family, fixed data distribution). Our universal formula is derived from seven diverse image-classification tasks, including binary classification (CelebA-Gender), fine-grained recognition (ImageNette), digit recognition (MNIST/SVHN), object recognition (CIFAR-10/100), and scene classification (Places365). The positive LODO $R^{2}$ across all seven confirms a degree of universality not demonstrated by prior work in the vision domain.

#### 8.2.3. Dual-Measure Correlation Screening

The combination of dCor and MIC provides a more complete picture of feature relevance than either Pearson or Spearman correlation alone. Specifically, MIC identified prm__lr as the top feature, while dCor ranked prm__batch first, a discrepancy that reveals qualitatively different types of dependence (non-monotonic vs. global). Single-measure correlation studies would miss this nuance.

#### 8.2.4. Statistical Rigor

Full BH-FDR correction across all 231 feature–dataset–epoch combinations prevents the false discovery rates that afflict uncorrected correlation studies. The analytical *t*-test for dCor and permutation test for MIC provide valid *p*-values without assuming Gaussianity.

#### 8.2.5. Complementary Accuracy and Latency Applications

A distinctive feature of our work is the repeated application of symbolic regression with PySR to two complementary edge-AI questions: accuracy prediction from training hyperparameters and architecture descriptors (Section 5, Section 6 and Section 7), and device-specific latency prediction from compute-oriented features (Section 6). The two pipelines share the PySR search procedure and interpretability criterion but use different features, targets, datasets, and validation protocols; they are not jointly optimized or combined into a single multi-objective formula. Existing latency prediction methods such as nn-Meter [30] and BRP-NAS [57] are designed exclusively for latency and produce neither accuracy formulas nor interpretable symbolic expressions. Hardware-aware NAS methods [31,32] integrate latency into architecture search but require device profiling and produce black-box lookups rather than equations a practitioner can inspect or reason about. Our contribution is to show that closed-form symbolic formulas are feasible for both objectives under these complementary setups, giving practitioners auditable surrogates for screening accuracy and latency separately before committing to full training or device profiling.

## 9. Discussion

### 9.1. Interpretation of Correlation Findings

The dual-measure screening with dCor and MIC reveals a nuanced picture that neither measure alone would expose. Batch size ranking first by dCor (0.228) but only seventh by MIC (0.125) indicates that its relationship with accuracy is predominantly monotonic and global: larger batches consistently shift accuracy in a predictable direction across all datasets. Learning rate, by contrast, ranks first by MIC (0.226) but third by dCor (0.170), consistent with its known U-shaped optimum: both very small and very large learning rates reduce accuracy, a non-monotonic pattern that MIC detects and dCor partially misses. This complementarity confirms that single-measure correlation screening is insufficient for hyperparameter analysis in diverse multi-dataset settings.

The dataset-dependence of feature importance carries direct implications for practitioners. For complex multi-class tasks (CIFAR-100, Places365), architectural features dominate; capacity and depth are the primary design levers. For simpler tasks (MNIST, SVHN, CelebA-Gender), training hyperparameters dominate; the architecture is largely irrelevant once a minimum threshold is exceeded. Practitioners deploying models on edge hardware can use this taxonomy to focus their optimization budget: architectural search for complex recognition tasks, hyperparameter tuning for simpler ones.

### 9.2. Scope and Limitations of Symbolic Formulas

The per-dataset symbolic formulas achieve $R^{2}$ values between 0.20 and 0.45, which is modest in absolute terms. These values reflect genuine epistemic limits: accuracy also depends on random initialization, weight trajectories, and data ordering, sources of variance not captured by the eleven features we analyze. A higher $R^{2}$ would require either finer-grained features (gradient norms, loss landscape curvature) or more training runs per configuration. Importantly, the primary contribution of this work is not predictive accuracy in isolation but interpretable, auditable structure: closed-form formulas that practitioners can inspect, reason about, and deploy at negligible per-query cost. Modest $R^{2}$ should therefore be judged against that interpretability objective rather than against opaque ensembles that maximize variance explained on a fixed feature basis. The formulas are best interpreted as ranking surrogates rather than precise regressors: they reliably distinguish high-accuracy configurations from low-accuracy ones, but should not be used to predict exact accuracy values without affine recalibration (Section 7.3).

A critical deployment restriction applies to EfficientNet-like and MobileNet-like architectures: the predictive sign reverses on both families (r=−0.47 and −0.29, respectively), meaning the formula ranks architectures in the wrong order for these families. This reversal is structural: it stems from the fact that these architectures achieve high accuracy through width scaling and depthwise separable convolutions rather than through depth scaling, violating the depth-as-capacity assumption embedded in the discovered formulas. Practitioners must exclude these families or apply family-specific recalibration before using the formulas for screening.

The universal formula’s LODO $R^{2}$ of 0.23 is intentionally modest: the primary objective is robust cross-dataset generalization, not maximum in-sample fit. The formula achieves strictly positive $R^{2}$ on every held-out dataset, a property that gradient boosting and linear regression both fail to maintain, making it a reliable screening surrogate even under distribution shift.

#### 9.2.1. Epoch Generalization Gap

As reported in Section 7.5, extrapolating from early to late training is difficult: test $R^{2}$ falls from 0.258 (trained on epochs 1 and 5) to 0.043 at epoch 50. The log-epoch term partially explains monotonic improvement but cannot capture path-dependent late-training dynamics; the magnitude of the gap reinforces that formulas should be used epoch-conditionally rather than as temporal extrapolators.

#### 9.2.2. Family-Held-Out Generalization

The LODO protocol (Section 7.4) holds out one dataset at a time but does not hold out one architecture family at a time. A family-held-out evaluation would test a stronger form of generalization: whether the formula’s ordering of, say, Transformer-like architectures can be predicted from training data containing only ResNet-like and VGG-like records. This is left to future work because several families have fewer than 100 records when isolated, making per-family LODO unreliable.

#### 9.2.3. Dataset Coverage

All seven datasets are image-classification tasks. Extension to object detection, segmentation, and generation would require additional feature engineering specific to those task types, and the current formulas should not be applied outside the classification setting without re-derivation.

### 9.3. Limitations of Inference Latency Models

Although the latency measurements span a range of input resolutions, all measurements were collected on the CIFAR-10 task. We explicitly tested whether the resulting architecture–latency relationships are confounded by input size (Section 6.6) and found resolution and architecture to be close to statistically independent (all VIF ≤1.25; distance correlation ≤0.141). Architectural features remain strongly predictive (log-space $R^{2}=0.62$–0.87) when input resolution is held physically fixed. The *H*-dependence captured by the CPU formula (Equation (25)) is therefore a genuine architectural effect rather than a resolution artifact. Generalization to other input resolutions (e.g., 224×224 ImageNet inputs) or sensor modalities (thermal, LiDAR) has not been validated, and validating the formulas on additional vision tasks at matched resolutions is left to future work. Additionally, the hardware measurements reflect a specific system configuration; latency on different hardware generations or under different thermal and power states may deviate from the derived formulas.

## 10. Conclusions

This paper presented a systematic, data-driven framework for understanding how architectural features and training hyperparameters determine the accuracy and inference latency of deep vision models deployed on edge hardware. By combining distance correlation and maximal information coefficient screening with PySR symbolic regression, we derived compact, interpretable mathematical formulas from diverse experimental data across seven image-classification benchmarks, with complementary device-specific latency formulas validated on CPU, GPU, and NPU hardware units representative of real edge deployment targets. Our key findings are:

1. **The joint use of dCor and MIC constitutes, to the best of our knowledge, the first dual-measure nonparametric correlation screening applied to this problem.** Batch size and total layer count are the most universally predictive features by dCor (0.228 and 0.174, respectively), while learning rate achieves the highest MIC (0.226), indicating a strongly non-monotonic relationship with accuracy that Spearman correlation would partially capture but dCor would underestimate—a nuance invisible to single-measure analyses.
2. **Feature–accuracy relationships are dataset-dependent.** Complex multi-class recognition datasets such as CIFAR-100 have accuracy primarily determined by architectural features (layer count, FLOPs, depth, parameter count), while simpler classification tasks such as CelebA-Gender, MNIST, and SVHN are dominated by hyperparameters (learning rate, batch size, dropout count). This distinction has direct practical implications for practitioners targeting edge hardware deployment: for complex recognition tasks on large datasets, architecture search matters most; for simpler tasks, hyperparameter tuning is the key lever.
3. **Symbolic regression, applied here for the first time to cross-dataset vision model accuracy prediction, produces human-readable formulas** with $R^{2}$ up to 0.45 per dataset and mean LODO $R^{2}=0.23$ for the universal model. The universal formula (Equation (27)) can be applied directly as a z-scored ranking surrogate, with optional affine recalibration (Section 7.3) when direct-units accuracy estimates are required.
4. **The universal formula generalizes positively to all seven held-out datasets**, making a defensible universality claim for vision model accuracy prediction, subject to the epoch-conditioning caveat from Section 7.5. Ordinary linear regression on the same features collapses to $R^{2}=-0.71$ on the most challenging held-out dataset, whereas the symbolic formula remains positive on every held-out dataset, confirming its robustness under domain shift.
5. **Inference latency on edge hardware is fundamentally device-heterogeneous and non-linear.** The device-specific symbolic formulas for GPU, NPU, and CPU (Equations (23)–(25)), representing the first closed-form empirically grounded latency formulas derived from a diverse neural architecture corpus, outperform all classical baselines, including FLOPs-linear regression, PALEO [13], and the Roofline model [14], by 6.7×–14.8× in $R^{2}$. A single pooled formula achieves negative $R^{2}$ on millisecond predictions, formally confirming that latency cannot be captured by a device-agnostic model. Together with the accuracy formulas, these results provide complementary interpretable surrogates for the two decisions central to edge hardware deployment: accuracy screening and per-device latency screening.

These results offer practical guidance for edge hardware designers: batch size and layer count should be primary design knobs when targeting a specific accuracy level under the memory and compute constraints of a deployment platform, and the universal formula is a quick screening tool for shortlisting candidate architectures before committing to full training runs. Future work will extend the latency formulas to additional vision tasks, testing whether architectural latency drivers generalize across input resolutions, analogous to the LODO evaluation performed for accuracy. Multi-objective symbolic regression that jointly optimizes accuracy and latency under a Pareto criterion is a natural extension toward automated, interpretable model selection for edge hardware deployment.

### Practical Guidance

The universal formula (Equation (27)) serves as a low-cost screening tool: given an architecture’s layer count *L*, batch size *B*, and target epoch *e*, it returns a z-scored accuracy estimate that ranks candidate configurations before committing to full training. Dataset-specific formulas (Section 5.2) give sharper per-task estimates when the target dataset is in scope. For inference latency, the device-specific formulas of Section 6 provide closed-form estimates appropriate to each hardware target. Practitioners should treat these formulas as decision-support surrogates: an affine recalibration (Section 7.3) on a small validation set converts the formula’s ranking signal into a direct-units accuracy estimate.

## Figures and Tables

**Table 1:** Feature descriptions. Architectural features (prefix nn_) characterize the static network graph; training hyperparameters (prefix prm__) describe the optimization configuration.

Feature	Type	Description
nn_total_params	Continuous	Total number of trainable parameters
nn_flops	Continuous	Forward-pass FLOPs
nn_total_layers	Continuous	Total number of layers
nn_max_depth	Continuous	Maximum sequential depth (longest path)
nn_has_attention	Binary	Presence of attention mechanism
nn_has_residual	Binary	Presence of residual connections
nn_dropout_count	Integer	Number of dropout layers
prm__lr	Continuous	Learning rate
prm__batch	Integer	Batch size
prm__dropout	Continuous	Dropout probability
prm__momentum	Continuous	Optimizer momentum

**Table 2:** Three-stage PySR pipeline. Progression to Stage 3 is conditional on sufficient $R^{2}$ improvement, preventing overfitting in the symbolic search.

Stage	Features Used	Condition
Stage 1	Top 3 by dCor	Always run
Stage 2	Top 5 by dCor	Always run
Stage 3	All significant features	Only if $R^{2}$ gain Stage 1 → 2 ≥0.05

**Table 3:** Input features for inference latency estimation formulas. All features are derived from neural network architecture specifications and the execution environment.

Symbol	Definition
$I_{p}$	Input pixels =H×W×C
log(*I_p_*)	Natural logarithm of input pixels
*F*	FLOPs in millions
$F_{\log}$	log(*F*): natural log of FLOPs in millions
*P*	Model parameters in millions
$P_{\log}$	log(*P*): natural log of parameters in millions
*P*_fp32_	Precision flag: 1 if FP32, 0 if INT8
*L*	Total number of layers
*H*	Input height (pixels)
*W*	Input width (pixels)
*C*	Input channels

**Table 4:** Mean distance correlation and Spearman |ρ| per feature across all 21 dataset–epoch combinations. All features show non-zero dependence.

Rank	Feature	Mean dCor	Mean |Spearmanρ|
1	prm__batch	**0.228**	0.206
2	nn_total_layers	0.174	0.166
3	prm__lr	0.170	0.312
4	nn_total_params	0.168	0.182
5	nn_max_depth	0.168	0.139
6	nn_dropout_count	0.164	0.149
7	nn_flops	0.127	0.176
8	nn_has_residual	0.094	0.086
9	prm__momentum	0.072	0.056
10	nn_has_attention	0.072	0.067
11	prm__dropout	0.066	0.032

**Table 5:** Mean MIC per feature across all 21 dataset–epoch combinations. The “Sig. tests” column shows how many of the 21 tests reached BH-FDR significance (α=0.05, permutation *p*-values with 200 shuffles). Bold indicates the highest mean MIC.

Rank	Feature	Mean MIC	Mean |Spearmanρ|	Sig. Tests
1	prm__lr	**0.226**	0.312	21/21
2	nn_total_params	0.204	0.182	19/21
3	nn_total_layers	0.187	0.166	19/21
4	nn_flops	0.186	0.176	20/21
5	nn_dropout_count	0.169	0.149	20/21
6	nn_max_depth	0.137	0.139	15/21
7	prm__batch	0.125	0.206	15/21
8	prm__dropout	0.119	0.032	5/21
9	prm__momentum	0.111	0.056	7/21
10	nn_has_residual	0.098	0.086	5/21
11	nn_has_attention	0.067	0.067	5/21

**Table 6:** Dataset-specific symbolic regression summary. L= nn_total_layers; F= nn_flops; D= nn_max_depth; P= nn_total_params; B= prm__batch; r= prm__lr; k= nn_dropout_count; R= nn_has_residual; attn = nn_has_attention. Bold indicates the best $R^{2}$.

Dataset	Best Stage (Features)	Best $R^{2}$	Top Feature (dCor)
CIFAR-10	Stage 2 (L,F,D,P,B)	**0.45**	nn_total_layers
CelebA-Gender	Stage 1 (B,r,k)	0.33	prm__batch
MNIST	Stage 1 (r,L,F)	0.28	prm__lr
CIFAR-100	Stage 1 (L,D,R)	0.28	nn_total_layers
ImageNette	Stage 2 (L,B,attn,k,F)	0.24	nn_total_layers
SVHN	Stage 1 (r,k)	0.23	prm__lr
Places365	Stage 1 (R,L,D)	0.20	nn_has_residual

**Table 7:** Robustness summary for the dataset-specific symbolic formulas of Section 5.2.1, Section 5.2.2, Section 5.2.3, Section 5.2.4, Section 5.2.5, Section 5.2.6 and Section 5.2.7. Best $R^{2}$ (train) is the training-fold maximum reported in Table 6. Val $R^{2}$ is the mean over the 5 cross-validation folds at the iteration count selected by convergence analysis (Appendix A). CI: 95% bootstrap percentile interval on the validation $R^{2}$ from B=1000 row-level resamples [51]. All datasets satisfy the overfitting budget Gap ≤0.05.

Dataset	Best $R^{2}$ (Train)	Val $R^{2}$ (5-Fold CV)	95% CI	Gap
CIFAR-10	0.47	0.43	[0.41,0.45]	0.04
CelebA-Gender	0.35	0.31	[0.29,0.33]	0.04
CIFAR-100	0.30	0.26	[0.23,0.29]	0.04
ImageNette	0.26	0.22	[0.19,0.25]	0.04
MNIST	0.31	0.27	[0.24,0.30]	0.04
Places365	0.22	0.18	[0.13,0.23]	0.04
SVHN	0.26	0.22	[0.20,0.24]	0.04

**Table 8:** Symbolic regression complexity vs. test $R^{2}$ for the selected best equations per device. Complexity is the PySR node count of the selected formula.

Device	Complexity	Test $R^{2}$ (log)	Test $R^{2}$ (ms)	RMSE (ms)
GPU	9	0.7366	0.4192	77.90
NPU	9	0.6697	0.4781	66.56
CPU	13	0.6055	0.4944	133.16
Pooled	5	0.3028	−0.0212	—

**Table 9:** Test $R^{2}$ scores (milliseconds): baseline methods vs. symbolic regression. Negative $R^{2}$ indicates predictions worse than the test-set mean. Best result per device is **bold**.

Method	CPU	GPU	NPU
*Baseline methods*			
B1: FLOPs-only linear [29]	0.0335	0.0627	0.0616
B2: FLOPs + MAC + Precision [30]	0.0335	0.0627	0.0616
B3: PALEO efficiency model [13]	−0.4207	−0.0823	−0.0779
B4: Roofline analytical model [14]	−0.4197	−0.0834	−0.0797
*Our method*			
Symbolic regression (PySR) [50]	**0.4944**	**0.4192**	**0.4781**

**Table 10:** Absolute and relative improvement of symbolic regression over the best-performing baseline (B1/B2) per device.

Device	Baseline $R^{2}$	Our $R^{2}$	$\Delta R^{2}$	Multiplier
CPU	0.0335	0.4944	+0.4609	14.8×
GPU	0.0627	0.4192	+0.3565	6.7×
NPU	0.0616	0.4781	+0.4165	7.8×

**Table 11:** Hardware specifications used for the Roofline baseline (B4). Values represent nominal peak performance.

Device	Peak Performance	Memory Bandwidth
CPU	350 GFLOPS	50 GB/s
GPU	5000 GFLOPS	400 GB/s
NPU	1000 GFLOPS	100 GB/s

**Table 12:** Collinearity between input resolution and architectural features (corpus level, n=1161). Values are computed once on the shared feature matrix and apply to all three devices. All measures indicate near-independence; VIF is far below the conventional 5–10 multicollinearity threshold.

$\log(I_{p})$ vs.	Pearson	Spearman	dCor
$F_{\log}$	0.137	0.103	0.141
$P_{\log}$	0.005	−0.007	0.051
*L*	0.155	0.019	0.134
*Variance-inflation factors (full feature set):*			
$\log(I_{p})=1.035$, $F_{\log}=1.247$, $P_{\log}=1.109$, L=1.186, $P_{\mathrm{fp}32}=1.000$			

**Table 13:** Fixed-resolution refit of the latency formulas (Section 6.6, analysis (iii)). Device formulas refit at a single fixed input resolution under the Section 3.6 PySR configuration. Log-space $R^{2}$ is the primary metric. Millisecond $R^{2}$ is shown where stable; at 64×64 the CPU value is suppressed by the back-transform of Equation (13) on a compressed dynamic range and is omitted.

Device	Resolution	*n*	Test $R^{2}$ (log)	Test $R^{2}$ (ms)
CPU	32×32	216	0.865	0.721
GPU	32×32	216	0.621	0.258
NPU	32×32	216	0.713	0.517
CPU	64×64	599	0.848	—
GPU	64×64	599	0.692	0.268
NPU	64×64	599	0.694	0.303

**Table 14:** Per-dataset calibration of the universal formula (Equation (27)) on the LEMUR 2 corpus [44]. $R_{\mathrm{cal}}^{2}=r^{2}$ is the variance explained by the best affine recalibration $\tilde{A}\approx \hat{\alpha }\;f+\hat{\beta }$. CIs: 95% bootstrap percentile intervals (B=1000 resamples). $\hat{\alpha }$, $\hat{\beta }$: slope and intercept of the recalibration. Bold indicates the pooled (overall) result.

Dataset	*n*	$R_{\mathrm{cal}}^{2}$	95% CI	Pearson *r*
CelebA-Gender	6089	0.104	[0.090,0.119]	0.322
CIFAR-10	17,449	0.046	[0.040,0.053]	0.215
CIFAR-100	5782	0.618	[0.595,0.640]	0.786
ImageNette	5446	0.468	[0.444,0.493]	0.684
MNIST	6118	0.050	[0.043,0.059]	0.225
Places365	723	0.078	[0.045,0.117]	0.279
SVHN	8163	0.166	[0.150,0.184]	0.408
**Pooled**	**49,770**	**0.130**	[0.124,0.136]	0.361

**Table 15:** Summary of the two LODO protocols. Means are *not* comparable across rows because the metric and training differ.

ID	What Is Evaluated?	Metric	Mean $R^{2}$
LODO-A	Fresh PySR on 6 datasets → test on 7th	PySR test $R^{2}$	0.231
LODO-B	Fixed Equation (27) vs. sklearn	$R_{\mathrm{cal}}^{2}$ for *all* methods	0.22 (formula)

**Table 16:** **LODO-A** (Table 15): per-fold PySR re-discovery. A new symbolic equation is fit on the six training datasets each fold; values are PySR test $R^{2}$ on the held-out dataset. **Not** the fixed Equation (27) (see LODO-B, Table 17). All seven folds are positive. Bold indicates the highest held-out $R^{2}$ and the mean.

Held-Out Dataset	LODO Test $R^{2}$
CIFAR-100	**0.365**
ImageNette	0.347
CIFAR-10	0.271
Places365	0.197
MNIST	0.195
SVHN	0.130
CelebA-Gender	0.112
**Mean**	**0.231**

**Table 17:** **LODO-B** (Table 15): head-to-head held-out $R_{\mathrm{cal}}^{2}$ per dataset and method (one metric for all columns). Equation (27): fixed, no retraining (matches Table 14; **not** LODO-A). Linear–GBM: fit on six training datasets, random_state =42. **Bold**: best interpretable method per row. ^a^ Raw sklearn test $R^{2}$ for CIFAR-10 Linear/Ridge is −0.71 (scale mismatch under domain shift); $R_{\mathrm{cal}}^{2}$ is reported here for fair comparison.

Held-Out	Equation (27)	Linear	Ridge	RF	GBM
CIFAR-100	**0.62**	0.49	0.49	0.59	0.60
ImageNette	**0.47**	0.39	0.39	0.56	0.57
CelebA-Gender	0.10	**0.20**	**0.20**	0.28	0.29
CIFAR-10	**0.05**	0.00 ^a^	0.00 ^a^	0.22	0.25
Places365	0.08	**0.11**	**0.11**	0.22	0.20
MNIST	0.05	**0.08**	**0.08**	0.22	0.23
SVHN	0.17	**0.20**	**0.20**	0.31	0.33
**Mean**	**0.22**	0.21	0.21	0.34	0.35

**Table 18:** Epoch generalization results. The formula trained on epochs {1,5} is evaluated on the held-out epoch 50. Bold indicates the held-out (test) result.

Split	$R^{2}$
Train (epochs 1, 5)	0.258
Test (epoch 50)	**0.043**

**Table 19:** Pooled-corpus comparison of the universal formula (Equation (27)) against four non-symbolic baselines restricted to the same feature basis. $R_{\mathrm{CV}}^{2}$: mean 5-fold cross-validation $R^{2}$; $R_{\mathrm{test}}^{2}$: held-out test $R^{2}$ from a single 70/15/15 split (random_state =42). Seed SD: standard deviation of $R_{\mathrm{CV}}^{2}$ across 10 random seeds (0–9). **Bold**: best interpretable method. ✔ denotes an interpretable method; × denotes a non-interpretable one.

Method	Interp.?	$R_{\mathrm{CV}}^{2}$	$R_{\mathrm{test}}^{2}$	Seed SD
Equation (27) (formula)	✔	0.130	0.139	—
**Linear**	✔	**0.328**	**0.327**	$9.2\times 10^{-5}$
Ridge (λ=1)	✔	**0.328**	**0.327**	$9.2\times 10^{-5}$
RF	×	0.503	0.508	$8.8\times 10^{-4}$
GBM	×	0.511	0.514	$7.6\times 10^{-4}$

**Table 20:** Comparison of accuracy and performance modeling approaches. NAS = Neural Architecture Search. Bold rows indicate the contributions of this work.

Approach	Formula Type	Task Scope	Interp.?	Data-Driven?
Kaplan et al. [6]	Power law (loss)	Language models	Yes	Yes
He et al. [16]	Architectural analysis	Single CV dataset	Partial	Empirical
NAS surrogates [8]	Black-box regressor	Multi-task	No	Yes
Roofline [14]	Analytical formula	Single device	Yes	No
PALEO [13]	Efficiency scalar	Single device	Yes	Partial
nn-Meter [30]	Linear regression	Multi-device	Partial	Yes
**This work (accuracy)**	**Symbolic (closed-form)**	**7 CV datasets**	**Yes**	**Yes**
**This work (latency)**	**Symbolic (closed-form)**	**CPU/GPU/NPU**	**Yes**	**Yes**

**Figure A1:**
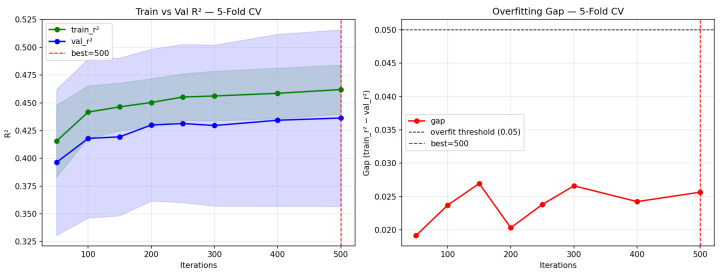
Convergence analysis for CIFAR-10: train $R^{2}$ (green) and validation $R^{2}$ (blue) as a function of PySR iteration count. The red dashed vertical line marks the selected iteration count (500); the shaded bands around each curve denote ±1 standard deviation across the five cross-validation folds.

**Figure A2:**
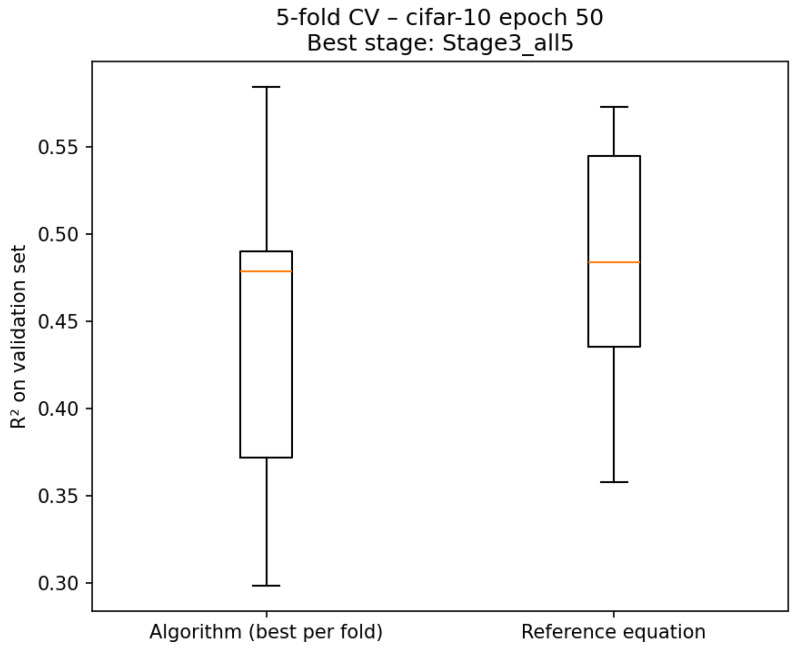
Five-fold CV boxplot for CIFAR-10 at epoch 50, comparing the algorithm (fold-specific best) and reference equation (global best) validation $R^{2}$. The near-equivalent performance of the reference equation across folds (low variance) confirms that the selected best equation generalizes stably.

## Data Availability

The neural network architectures and training records used in this study are available via the LEMUR 2 corpus [44] and the nn-dataset repository at https://github.com/abrain-one/nn-dataset (accessed on 1 November 2025). Inference latency measurements and analysis scripts are available at https://github.com/ABrain-One/nn-stat (accessed on 1 November 2025).

## Abbreviations

The following abbreviations are used in this manuscript:

dCor	Distance Correlation
MIC	Maximal Information Coefficient
PySR	Python Symbolic Regression
NAS	Neural Architecture Search
FLOPs	Floating-Point Operations (count)
LODO	Leave-One-Dataset-Out
BH-FDR	Benjamini–Hochberg False Discovery Rate
FP32	32-bit Floating Point
INT8	8-bit Integer
CPU	Central Processing Unit
GPU	Graphics Processing Unit
NPU	Neural Processing Unit
CV	Cross-Validation

## Appendix A. Convergence Analysis

### Appendix A.1. Motivation

The number of PySR iterations controls the quality of the symbolic search: too few iterations result in suboptimal equations; too many can lead to overfitting (discovering spurious patterns present only in the training fold). To select the optimal iteration count per dataset, we performed a five-fold cross-validation convergence analysis.

### Appendix A.2. Procedure

For each dataset and epoch 50, we ran the full three-stage PySR pipeline at iteration counts {50,100,150,200,250,300,400,500}, each evaluated on five cross-validation folds. For each fold, the best-stage model’s training $R^{2}$ and validation $R^{2}$ were recorded. We report mean ± standard deviation across folds and the overfitting gap (train $R^{2}$ − val $R^{2}$).

The selected iteration count for each dataset was the value maximizing mean validation $R^{2}$ while keeping the overfitting gap below 0.05. Table A1 reports the selected counts, and Figure A1 illustrates the convergence behavior for CIFAR-10.

### Appendix A.3. Results

**Table A1:** Selected iteration counts from convergence analysis. Values rounded to nearest 0.01 for readability; actual values from convergence CSV files.

Dataset	Selected Iter.	Train $R^{2}$	Val $R^{2}$	Gap
CIFAR-10	500	0.47	0.43	0.04
CelebA-Gender	500	0.35	0.31	0.04
CIFAR-100	200	0.30	0.26	0.04
ImageNette	200	0.26	0.22	0.04
MNIST	200	0.31	0.27	0.04
Places365	50	0.22	0.18	0.04
SVHN	200	0.26	0.22	0.04

Places365 requires only 50 iterations due to its small epoch-50 sample size (n=87): more iterations produce increasingly overfitted equations on this limited data. The two largest datasets, CIFAR-10 and CelebA-Gender, benefit from 500 iterations, reflecting the richness of their respective feature–accuracy relationships.

### Appendix A.4. Extended Baseline Comparison Tables for Inference Latency

For completeness, we reproduce here the full per-device baseline comparison from Section 6.2, with both log-space and millisecond $R^{2}$ alongside RMSE in milliseconds. These extended results are presented in Table A2.

**Table A2:** Extended baseline comparison for inference latency: $R^{2}$ (log-space), $R^{2}$ (milliseconds), and RMSE (ms) for all four baselines and our symbolic regression formulas across all three devices. Negative $R^{2}$ indicates predictions worse than the test-set mean. MAPE is omitted because near-zero true latency on a small number of CPU and NPU test samples makes the percentage metric degenerate (values exceeding $10^{5}\%$); RMSE in milliseconds is reported as the unitful error metric instead. Bold indicates the best result per device.

**CPU**			
**Method**	$R^{2}$ **(log)**	$R^{2}$ **(ms)**	**RMSE (ms)**
B1: FLOPs-only linear	0.031	0.0335	183.81
B2: Multi-feature linear	0.031	0.0335	183.81
B3: PALEO	−0.12	−0.4207	222.86
B4: Roofline	−0.12	−0.4197	222.78
**PySR (ours)**	**0.6055**	**0.4944**	**133.16**
**GPU**			
Method	$R^{2}$ (log)	$R^{2}$ (ms)	RMSE (ms)
B1: FLOPs-only linear	0.052	0.0627	89.52
B2: Multi-feature linear	0.052	0.0627	89.52
B3: PALEO	−0.04	−0.0823	96.19
B4: Roofline	−0.04	−0.0834	96.24
**PySR (ours)**	**0.7366**	**0.4192**	**77.90**
**NPU**			
Method	$R^{2}$ (log)	$R^{2}$ (ms)	RMSE (ms)
B1: FLOPs-only linear	0.048	0.0616	92.91
B2: Multi-feature linear	0.048	0.0616	92.91
B3: PALEO	−0.04	−0.0779	99.58
B4: Roofline	−0.04	−0.0797	99.66
**PySR (ours)**	**0.6697**	**0.4781**	**66.56**

### Appendix A.5. CV Boxplots

For the final selected model per dataset, we show the distribution of per-fold $R^{2}$ values for both the algorithm’s best-per-fold equation and the reference (global best) equation. Figure A2 shows this distribution for CIFAR-10.
